# Multimodal deep learning improves recurrence risk prediction in pediatric low-grade gliomas

**DOI:** 10.1093/neuonc/noae173

**Published:** 2024-08-30

**Authors:** Maryamalsadat Mahootiha, Divyanshu Tak, Zezhong Ye, Anna Zapaishchykova, Jirapat Likitlersuang, Juan Carlos Climent Pardo, Aidan Boyd, Sridhar Vajapeyam, Rishi Chopra, Sanjay P Prabhu, Kevin X Liu, Hesham Elhalawani, Ali Nabavizadeh, Ariana Familiar, Sabine Mueller, Hugo J W L Aerts, Pratiti Bandopadhayay, Keith L Ligon, Daphne Haas-Kogan, Tina Y Poussaint, Hemin Ali Qadir, Ilangko Balasingham, Benjamin H Kann

**Affiliations:** Faculty of Medicine, University of Oslo, Oslo, Norway; The Intervention Centre, Oslo University Hospital, Oslo, Norway; Department of Radiation Oncology, Dana-Farber Cancer Institute, Brigham and Women’s Hospital, Boston Children’s Hospital, Harvard Medical School, Boston, Massachusetts, USA; Artificial Intelligence in Medicine (AIM) Program, Mass General Brigham, Harvard Medical School, Boston, Massachusetts, USA; Department of Radiation Oncology, Dana-Farber Cancer Institute, Brigham and Women’s Hospital, Boston Children’s Hospital, Harvard Medical School, Boston, Massachusetts, USA; Artificial Intelligence in Medicine (AIM) Program, Mass General Brigham, Harvard Medical School, Boston, Massachusetts, USA; Department of Radiation Oncology, Dana-Farber Cancer Institute, Brigham and Women’s Hospital, Boston Children’s Hospital, Harvard Medical School, Boston, Massachusetts, USA; Artificial Intelligence in Medicine (AIM) Program, Mass General Brigham, Harvard Medical School, Boston, Massachusetts, USA; Department of Radiation Oncology, Dana-Farber Cancer Institute, Brigham and Women’s Hospital, Boston Children’s Hospital, Harvard Medical School, Boston, Massachusetts, USA; Artificial Intelligence in Medicine (AIM) Program, Mass General Brigham, Harvard Medical School, Boston, Massachusetts, USA; Department of Radiation Oncology, Dana-Farber Cancer Institute, Brigham and Women’s Hospital, Boston Children’s Hospital, Harvard Medical School, Boston, Massachusetts, USA; Artificial Intelligence in Medicine (AIM) Program, Mass General Brigham, Harvard Medical School, Boston, Massachusetts, USA; Department of Radiation Oncology, Dana-Farber Cancer Institute, Brigham and Women’s Hospital, Boston Children’s Hospital, Harvard Medical School, Boston, Massachusetts, USA; Artificial Intelligence in Medicine (AIM) Program, Mass General Brigham, Harvard Medical School, Boston, Massachusetts, USA; Department of Radiation Oncology, Dana-Farber Cancer Institute, Brigham and Women’s Hospital, Boston Children’s Hospital, Harvard Medical School, Boston, Massachusetts, USA; Artificial Intelligence in Medicine (AIM) Program, Mass General Brigham, Harvard Medical School, Boston, Massachusetts, USA; Department of Radiology, Boston Children’s Hospital, Harvard Medical School, Boston, Massachusetts, USA; Department of Radiation Oncology, Dana-Farber Cancer Institute, Brigham and Women’s Hospital, Boston Children’s Hospital, Harvard Medical School, Boston, Massachusetts, USA; Artificial Intelligence in Medicine (AIM) Program, Mass General Brigham, Harvard Medical School, Boston, Massachusetts, USA; Department of Radiology, Boston Children’s Hospital, Harvard Medical School, Boston, Massachusetts, USA; Department of Radiation Oncology, Dana-Farber Cancer Institute, Brigham and Women’s Hospital, Boston Children’s Hospital, Harvard Medical School, Boston, Massachusetts, USA; Department of Radiation Oncology, Dana-Farber Cancer Institute, Brigham and Women’s Hospital, Boston Children’s Hospital, Harvard Medical School, Boston, Massachusetts, USA; Department of Radiology, Perelman School of Medicine, University of Pennsylvania, Philadelphia, Pennsylvania, USA; Center for Data-Driven Discovery in Biomedicine (D3b), Children’s Hospital of Philadelphia, Philadelphia, Pennsylvania, USA; Department of Neurosurgery, Children’s Hospital of Philadelphia, Philadelphia, Pennsylvania, USA; Center for Data-Driven Discovery in Biomedicine (D3b), Children’s Hospital of Philadelphia, Philadelphia, Pennsylvania, USA; Department of Neurological Surgery, University of California San Francisco, San Francisco, California, USA; Department of Pediatrics, University of California San Francisco, San Francisco, California, USA; Department of Neurology, University of California San Francisco, San Francisco, California, USA; Radiology and Nuclear Medicine, CARIM & GROW, Maastricht University, Maastricht, The Netherlands; Department of Radiology, Brigham and Women’s Hospital, Dana-Farber Cancer Institute, Harvard Medical School, Boston, Massachusetts, USA; Department of Radiation Oncology, Dana-Farber Cancer Institute, Brigham and Women’s Hospital, Boston Children’s Hospital, Harvard Medical School, Boston, Massachusetts, USA; Artificial Intelligence in Medicine (AIM) Program, Mass General Brigham, Harvard Medical School, Boston, Massachusetts, USA; Department of Pediatric Oncology, Dana-Farber Cancer Institute, Boston Children’s Hospital, Harvard Medical School, Boston, Massachusetts, USA; Department of Pathology, Dana-Farber Cancer Institute, Boston Children’s Hospital, Harvard Medical School, Boston, Massachusetts, USA; Department of Radiation Oncology, Dana-Farber Cancer Institute, Brigham and Women’s Hospital, Boston Children’s Hospital, Harvard Medical School, Boston, Massachusetts, USA; Department of Radiology, Boston Children’s Hospital, Harvard Medical School, Boston, Massachusetts, USA; The Intervention Centre, Oslo University Hospital, Oslo, Norway; Department of Electronic Systems, Norwegian University of Science and Technology, Trondheim, Norway; The Intervention Centre, Oslo University Hospital, Oslo, Norway; Department of Radiation Oncology, Dana-Farber Cancer Institute, Brigham and Women’s Hospital, Boston Children’s Hospital, Harvard Medical School, Boston, Massachusetts, USA; Artificial Intelligence in Medicine (AIM) Program, Mass General Brigham, Harvard Medical School, Boston, Massachusetts, USA

**Keywords:** cancer prognosis, deep learning, event-free survival, MRI, pediatric low-grade glioma

## Abstract

**Background:**

Postoperative recurrence risk for pediatric low-grade gliomas (pLGGs) is challenging to predict by conventional clinical, radiographic, and genomic factors. We investigated if deep learning (DL) of magnetic resonance imaging (MRI) tumor features could improve postoperative pLGG risk stratification.

**Methods:**

We used a pretrained DL tool designed for pLGG segmentation to extract pLGG imaging features from preoperative T2-weighted MRI from patients who underwent surgery (DL-MRI features). Patients were pooled from 2 institutions: Dana Farber/Boston Children’s Hospital (DF/BCH) and the Children’s Brain Tumor Network (CBTN). We trained 3 DL logistic hazard models to predict postoperative event-free survival (EFS) probabilities with (1) clinical features, (2) DL-MRI features, and (3) multimodal (clinical and DL-MRI features). We evaluated the models with a time-dependent Concordance Index (C^td^) and risk group stratification with Kaplan–Meier plots and log-rank tests. We developed an automated pipeline integrating pLGG segmentation and EFS prediction with the best model.

**Results:**

Of the 396 patients analyzed (median follow-up: 85 months, range: 1.5–329 months), 214 (54%) underwent gross total resection and 110 (28%) recurred. The multimodal model improved EFS prediction compared to the DL-MRI and clinical models (C^td^: 0.85 (95% CI: 0.81–0.93), 0.79 (95% CI: 0.70–0.88), and 0.72 (95% CI: 0.57–0.77), respectively). The multimodal model improved risk-group stratification (3-year EFS for predicted high-risk: 31% versus low-risk: 92%, *P* < .0001).

**Conclusions:**

DL extracts imaging features that can inform postoperative recurrence prediction for pLGG. Multimodal DL improves postoperative risk stratification for pLGG and may guide postoperative decision-making. Larger, multicenter training data may be needed to improve model generalizability.

**Key Points**

- Postoperative risk prediction for pLGG is challenging with traditional risk factors.
- Multimodal DL with imaging features improves postoperative recurrence risk prediction by 13%.
- Multimodal DL effectively stratifies patients into low-risk (92% EFS) and high-risk (31% EFS) groups.

**Importance of the Study**

Postoperative recurrence risk for pediatric low-grade gliomas (pLGGs) is challenging to predict due to their varied biology and unclear risk factors. Resultingly, optimal adjuvant management, balancing recurrence risk reduction against therapy toxicity, is challenging. This study advances recurrence prediction and risk stratification for pLGGs, introducing an automated, deep learning (DL) pipeline integrating preoperative MRI and clinical data. The model, validated across 2 distinct patient cohorts, utilizes a pretrained MRI feature extractor and supports a hands-free and practical workflow that can be applied to routinely acquired MRI. It effectively learns tumoral imaging features that portend recurrence risk and combines these with electronic health record-extracted clinical data for improved recurrence prediction. The resulting model stratifies patients into high- and low-risk groups with ~30% and >90% 3-year event-free survival, respectively. With further validation, DL pipelines that harness imaging and clinical data could help guide decisions regarding postoperative treatment and surveillance for pLGG.

Brain tumors are the most common type of solid tumor and cause of cancer-related death in children.^1^ Pediatric low-grade gliomas (pLGGs) are gradually progressing brain tumors that originate from glial cells and are categorized as World Health Organization grades I or II,^2^ accounting for 30–50 percent of all childhood central nervous system tumors.^3^ Collectively, pLGGs represent a basket of >20 histologies^4^ and, more recently defined, heterogeneous molecular characteristics,^5^ with nearly 50% of tumors harboring a BRAF-associated mutation.^6^

While pLGGs confer a relatively good prognosis following primary tumor resection compared to high-grade tumors, postoperative outcomes are heterogeneous. Patients undergoing surgical treatment with gross total resection (GTR) typically exhibit favorable outcomes, demonstrating over 85% progression-free survival, yet recurrences still occur.^7,8^ Conversely, the prognosis for patients who have undergone subtotal resections without subsequent adjuvant therapy is less frequently reported. Available literature indicates that in these cases, the progression rate ranges from 40% to 80%.^9,10^ The diverse progression patterns inherent in natural history add complexity to decisions regarding adjuvant therapy,^2^ a challenge further intensified with the advent of targeted treatments, particularly those for BRAF-associated mutations.^6^

While the histological and molecular diagnosis of tumors remains paramount in determining prognosis, therapy, and survival outcomes,^6^ the potential of magnetic resonance imaging (MRI) as a reliable source for prognostic information warrants exploration. MRI surveillance is standard postoperatively, though decisions regarding MRI surveillance interval and duration are challenging. Early identification of recurrence optimizes the prospects for effective second-line intervention and the preservation of the patient’s quality of life.^2,11^ Thus, frequent MRI surveillance, particularly in the early postoperative period, is standard across patients despite the very heterogeneous natural histories of pLGGs. These scans come with significant anxiety for patients and families, and cost,^12^ and better risk-stratification may provide an opportunity to tailor surveillance schedules to patient risk.

MRI provides crucial information about tumor size, location, and other characteristics that guide therapeutic decisions and follow-up management,^13–17^ though traditional imaging features, such as texture and contrast-enhancement have some limitations in prognosis.^13,14,17^ Recent studies have suggested that molecular and histological characteristics of brain tumors manifest in MR imaging features extracted quantitatively (ie, Radiomics).^15,18^ Radiomics may have utility in improving risk stratification for pLGG, though the field is in a nascent stage.^15^

Several investigations, mainly small, single-institutions studies, have been conducted for survival prediction in brain cancer, relying on traditional radiomic features, including texture and shape features rather than utilizing features derived from deep learning (DL).^19–24^ These models have been difficult to reproduce and validate externally, potentially owing to small datasets and the instability of radiomic features to perturbations in tumor segmentation and scanner parameters.^25^

More recently, deep-learning imaging analysis techniques have emerged and surpassed traditional radiomic studies in terms of performance and utility in multiple cancer applications.^26–28^ DL has been slow to be adopted in pediatric brain tumors, likely due to limited and sparse datasets. Recent advances in DL methodologies, such as transfer learning, self-supervised learning, and foundational models,^29^ may provide the basis for successful deep learning applications in pediatric brain tumor analysis.^30–32^ Furthermore, imaging data coupled with electronic health record (EHR)-derived patient and treatment characteristics (ie, multimodal learning) has been found to further improve model predictive performance.^32–34^

Our study aimed to predict recurrence in pLGG by employing a DL approach that integrates MRI imaging and clinical variables. We utilized a pretrained segmentation model for feature extraction to explore whether a knowledge transfer and multimodal learning strategy could enhance risk stratification and recurrence prediction in pLGG postoperatively using a multi-institutional dataset.

## Materials and Methods

### Datasets

This study was conducted in accordance with the Declaration of Helsinki guidelines and Institutional Review Board approval at participating institutions. A waiver of consent was obtained prior to research initiation. In this retrospective study, we analyzed data from 2 distinct pediatric glioma cohorts: Dana-Farber/Boston Children’s Hospital (DF/BCH) and the Children’s Brain Tumor Network (CBTN). DF/BCH included all patients with a pathologically confirmed diagnosis of WHO Grade I-II pLGG diagnosed from 1992 until 2019 who underwent a surgical procedure (biopsy, subtotal resection, and/or GTR) and had a preoperative, diagnostic quality MRI available. Data was collected and abstracted from the EHR from June 2022 to June 2023 by a trained clinical research assistant and reviewed by a board-certified radiation oncologist (B.H.K.). Patients with neurofibromatosis were excluded, given the distinct disease trajectory compared with other pLGGs.^35^ The CBTN cohort^36^ includes patients treated across several institutions, with the majority (*N* = 186, 95%) treated at the Children’s Hospital of Philadelphia.

A recurrence event was documented if any of the following criteria were met: (1) radiographic and/or clinical impression of recurrence as abstracted from the radiology report or neuro-oncology note (with or without pathologic confirmation), (2) new clinical symptom attributable to tumor, or (3) change in clinical management. If radiographic impression was equivocal for recurrence, then confirmation in a neuro-oncology note with clinical impression of recurrence and associated symptoms or management change would be required to document an event. Event-free survival (EFS), the study's primary endpoint, was defined by any recurrence event and/or death and was designated as the earliest date on which one of the events was observed.

**Figure 1.**
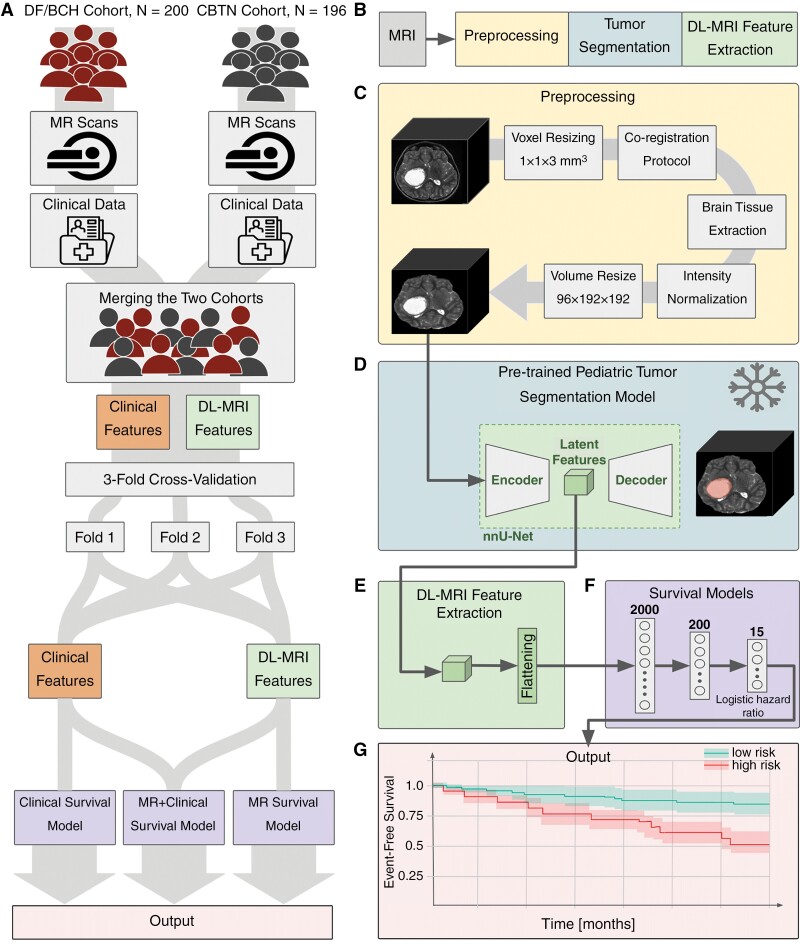
Study framework. (A) The whole framework of the study: DF/BCH and CBTN cohorts were merged, clinical features (EHR-derived clinical features for each patient that are normalized) and deep learning (DL)-magnetic resonance imaging (MRI) features were extracted. The data was split into a development set (*n* = 277) and a held-out test set (*n* = 119). The development set was utilized for 3-fold cross-validation. Three survival models were developed: One based on clinical features, one based on DL-MRI features, and one based on both (multimodal). (B) DL-MRI features are the outputs of 3 steps: preprocessing, tumor segmentation, and feature extraction. (C) Preoperative T2-weighted MRI sequences were preprocessed before tumor segmentation. (D) Preprocessed scans were input into a frozen pretrained pLGG segmentation nnUNet model from prior work^30^ to output tumor masks. This pretrained model was initially trained on BraTS and some CBTN subjects, but feature performance was consistent across both DF/BCH and CBTN cohorts. (E) Encoded features from the nnUNet model were flattened into a feature vector. (F) A 3-layer neural network survival model was trained via 3-fold cross-validation on the training set using a logistic hazard loss function to predict event-free survival (EFS). (G) The model outputs personalized EFS curves. Model evaluation was primarily based on C-index scores on the held-out test set. DF/BCH, Dana-Farber/ Boston Children’s Hospital; CBTN, children’s brain tumor network; MRI, magnetic resonance imaging; EHR, electronic health record; DL, deep learning; BraTS, brain tumor segmentation; EFS, event-free survival.

The same inclusion and exclusion criteria were used for the CBTN cohort, acquired under the Data Use Agreement, and last updated in October 2023 (Supplementary Figure S1). The preoperative MRI T2-weighted sequence for each patient that was closest to the surgery date was selected for analysis. In the DF/BCH dataset, the time from image acquisition to surgery ranged from 0 to 3611 days, with a median of 4 days and a mean of 80 days. In the CBTN dataset, this time ranged from 0 to 2153 days, with a median of 1 day and a mean of 19 days. The study framework is found in Figure 1. Further detail is found in Supplementary Material.

### MRI Preprocessing

MRI images were converted from DICOM format to NIFTI format via rasterization packages utilizing the dcm2nii package. N4 bias field correction was adopted to correct the low-frequency intensity non-uniformity present in MRI images using SimpleITK (SITK). All scans were resampled to 1 × 1 × 3 mm^3^ voxel size using linear interpolation and then coregistered with each other using rigid registration with SITK. Brain extraction was performed for all the scans using the HD-BET package in Python v3.8.

### MRI Feature Extraction

A pretrained, UNet-based pediatric glioma 3D tumor segmentation model for T2-weighted MRI that was developed and validated in prior work^30^ was applied to all scans. Of note is that the segmentation model had been previously fine-tuned on a subset of CBTN patients but not on any DF/BCH patients. Given the relatively small amount of training data available for pLGG, we hypothesized that training a DL network based on imaging features from scratch to predict EFS would be at high risk for overfitting. To overcome this problem, we used transfer learning. Specifically, we devised a technique to extract the feature set derived from the final layer of the encoder within the 3D segmentation model.^37^ These latent DL-MRI features, derived from the encoder, with a size of 4096 features, encapsulate high-level abstract representations of the tumor within the nnU-Net. [96, 192,192] was used as the input size for extracting features from the segmentation model. We hypothesized these features, which are optimized for tumor segmentation, inherently learn to recognize patterns of tumor voxel intensities and borders that would be informative when transferred to recurrence prediction.

### Event Free Survival (EFS) Prediction

We developed a fully automated pipeline, incorporating our pretrained segmentation algorithm, feature extractor, and a fully connected DL survival model for EFS prediction (Figure 1).

### DL Survival Model Architecture

We utilized the extracted feature vectors to train a 3-layer fully connected neural network, which was designed for EFS prediction. The survival analysis was conducted using a DL model grounded in the logistic hazard framework^38^ with a discrete-time model.^39^ The logistic hazard model treats survival analysis as serial binary logistic regressions for distinct time intervals, estimating event probability sequentially (Supplementary Material). We trained 3 models with identical architectures: (1) a model with EHR-derived variables, including age at diagnosis, and resection status (gross total, subtotal, and biopsy only) (2) a model with DL-MRI features alone, and (3) a model combining EHR and DL-MRI data (multimodal). For the clinical model, the noncategorical variable age was normalized using the mean and standard deviation. These clinical variables were treated as a feature vector and concatenated with the last fully connected layer of the survival network.

### Model Training and Validation

To maximize the number and representations of tumors available to the neural network, we pooled the DF/BCH and CBTN datasets for training and testing. We also observed notable differences in EFS rate, disease, and treatment characteristics between the cohorts (see Results), motivating us to pool data to create a more generalizable model. We randomly split the combined cohort, stratified by EFS status and data source: 70% for development and 30% for testing. We applied 3-fold cross-validation on the development set to evaluate the mean survival performance (Supplementary Material). Training details for the clinical, DL-MRI, and multimodal models are found in Supplementary Material. To determine if features extracted from one institution generalized to another, we also investigated 2 models trained separately on DF/BCH-only and CBTN-only data, respectively (Supplementary Material). Finally, we examined how fine-tuning the models on incrementally increasing amounts of data from the external institution would improve performance (Supplementary Material).

### Model Evaluation and Statistical Analysis

The primary study endpoint was discriminatory performance for EFS via the time-dependent concordance index (C-index).^40^ Bootstrapping was performed with 1000 resampling iterations to generate 95% confidence intervals for the C-indexes. Time-dependent receiver operating characteristic analysis with area under the curve (AUC) for 3-year EFS was also evaluated.^41^ Calibration was checked visually with plots to evaluate how closely the model’s predicted probabilities match the actual observed outcomes.^42^ The expected calibration error was calculated based on the discrepancy between these predicted probabilities and the observed proportions of nonrecurrence. Additionally, the integrated Brier score was evaluated for the Supplementary Material models, given its relevance as a robust metric for the assessment of survival predictions^43^ (). Differences between patient cohorts were analyzed with Fisher’s Exact test or Wilcoxon test, as appropriate. Kaplan–Meier survival curve comparisons were analyzed with log-rank tests. Individual clinical features for the clinical model were first evaluated with univariable Cox regression.^44^ Variables associated with EFS on univariable analysis (as defined a priori by a *P*-value < .15) were included in the multivariable clinical and multimodal prediction models (Supplementary Material). A two-sided *P*-value of .05 was considered statistically significant unless otherwise noted. Python v3.8 was utilized for data curation, DL, and survival analysis and statistical analysis was performed using libraries scikit-learn, sksurv, pycox, and lifelines. For advanced graphical representation, R was employed with ggplot2, gridExtra, vioplot, survival, survminer, and rms.

## Results

### Patient Characteristics and Risk of Recurrence

Of 396 patients included for analysis, 200 were from DF/BCH and 196 from CBTN (Table 1). Compared with the CBTN cohort, the DF/BCH cohort had slightly older age (median: 8.7 years vs 5.7 years, *P* < .01), a higher proportion of GTRs (61% vs 47%, *P* < .01), and a lower proportion of patients receiving adjuvant chemotherapy (5% vs 25%, *P* < .01). Three-year and overall event rates were higher in the CBTN cohort compared to DF/BCH (26% vs 11%, and 36% vs 19%, *P* < .01 for each, respectively). Actuarial 3-year EFS was 89% for DF/BCH and 69% for CBTN (Supplementary Figure S26). For DF/BCH, the median event time was 35.4 months (range: 1 to 203), and for CBTN, the median event time was 19.4 months (range: 2 to 201). All clinical characteristics were evenly balanced between model development and test sets (Supplementary Table S4). Regarding MRI parameters, slice thickness, echo time, repetition time, and flip angle were significantly different between DF/BCH and CBTN (Supplementary Table S5, Figure S24–S25).

**Table 1.** Comparative Demographics of Patients in the DF/BCH and CBTN Cohorts. The Event Includes Radiographic and/or Clinical Recurrence/Progression and/or Death

Characteristics	DF/BCH cohort (*N* = 200)	CBTN cohort (*N* = 196)	*P*-value
Age (years) range, median	[0.4,19], 8.7	[0.2,19.4], 5.7	<.01
Sex			
Male	112 (56 %)	101 (52 %)	.36
Female	88 (44 %)	95 (48 %)	
Resection status			
Gross total	122 (61 %)	92 (47 %)	<.01
Partial	59 (29 %)	64 (33 %)	
Biopsy	17 (9 %)	38 (19 %)	
Not available	2 (1 %)	2 (1 %)	
BRAF mutational status			
V600E	21 (10 %)	11 (6 %)	<.01
Fusion	19 (9 %)	47 (24 %)	
Wild-type	9 (5 %)	34 (17 %)	
Unknown	151 (76 %)	104 (53 %)	
BRAF mutational status (with unknowns inferred) ^*^			
V600E	45 (22 %)	33 (17 %)	
Fusion	77 (39 %)	74 (38 %)	<.01
Wild type	78 (39 %)	89 (45 %)	
Chemotherapy			
Yes	9 (5 %)	49 (25 %)	<.01
No	191 (95 %)	145 (74 %)	
Not available	0 (0 %)	2 (1 %)	
Radiotherapy			
Yes	7 (4 %)	15 (8 %)	.1
No	193 (96 %)	178 (91 %)	
Not available	0 (0 %)	3 (1 %)	
Tumor location			
Posterior fossa	77 (38%)	82 (42%)	<.01
Supratentorial	74 (37%)	35 (18%)	
Noncortical supratentorial	23 (12%)	30 (15%)	
Optic pathway/noncortical supratentorial	2 (1%)	19 (10%)	
Brainstem	21 (11%)	16 (8%)	
Spinal cord	3 (1%)	11 (6%)	
Other	0 (0 %)	3 (1%)	
3-year recurrence (*N*)	22 (11 %)	51 (26 %)	<.01
Total recurrence (*N*)	39 (19 %)	71 (36 %)	<.01
Recurrence time (days) range, median	[20, 6168], 1077	[76, 6119], 592	<.01

^*^BRAF mutational status was confirmed by genomic analysis on surgical tissue specimens in 255 patients. For the purposes of the study, we conducted a sensitivity analysis in which BRAF mutational status was inferred radiographically using a previously published, magnetic resonance imaging-based deep learning algorithm.^31^.

On univariable analysis, GTR and older age at diagnosis were associated with improved EFS (Supplementary Table S1). BRAF mutational status (V600E, fusion, or wild type), gender, receipt of chemotherapy or radiotherapy, and location of tumor were not significantly associated with EFS. Given that BRAF mutational status was only available for 35% of patients, we conducted a sensitivity analysis inferring unknown BRAF status with a previously validated imaging-based BRAF mutational status prediction tool^31^ (Supplementary Table S2), and BRAF status remained unassociated with EFS.

### Model Performance for Risk Prediction

**Figure 2.**
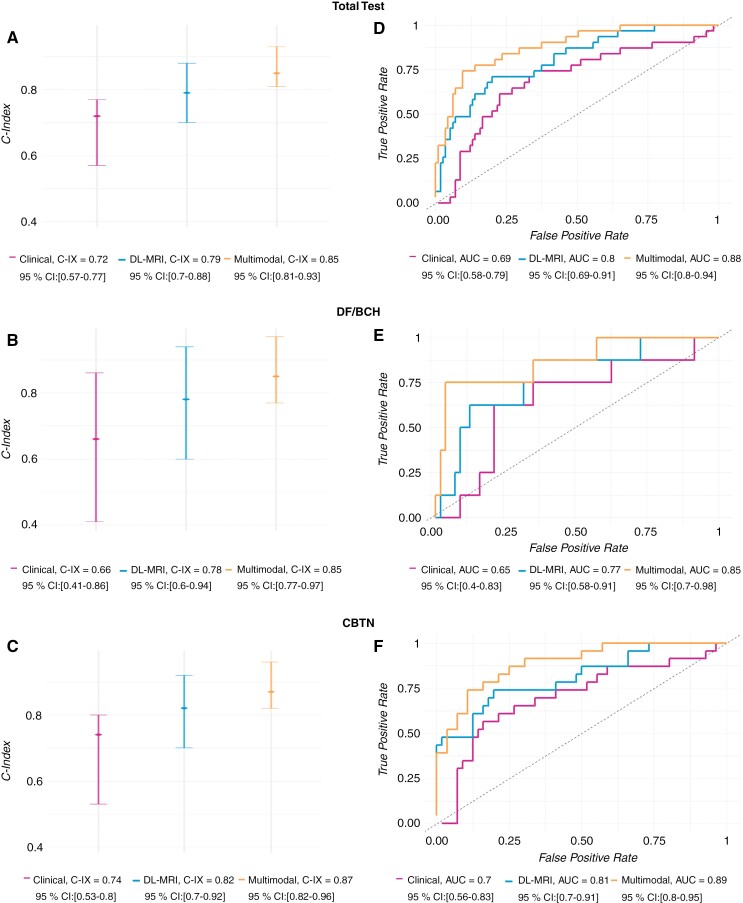
C-indexes and ROC curves for comparing model performance. The C-indexes with 95 % CI analyses consistently indicated the best model performance of the multimodal model among the 3 models, followed by the image model and clinical model for the entire test set (A), DF/BCH cohort (B), and CBTN cohort (C). The ROC analyses consistently indicated the best model performance of the multimodal model among the 3 models, followed by the image model and clinical model for the entire test set (D), DF/BCH cohort (E), and CBTN cohort (F). C-IX, C-Index.

On evaluation in the blinded test set (*n* = 119), the multimodal model (DL-MRI and clinical features) had the highest discriminatory performance (C-index: 0.85 [95% CI: 0.81–0.93]), followed by the DL-MRI model (C-index: 0.79 [95% CI: 0.70–0.88]), and lastly the clinical model (C-index: 0.72 [95% CI: 0.57–0.77]; Figure 2A). Performance of the multimodal model was stable across DF/BCH and CBTN test sets (0.85 [95% CI: 0.77–0.97] and 0.87 [95% CI: 0.82–0.96], respectively), while performance was less stable for the DL-MRI model (0.78 [95% CI: 0.60–0.94] and 0.82 [95% CI: 0.70–0.92], respectively) and the clinical model (0.66 [95% CI: 0.41–0.86] and 0.74 [95% CI: 0.53–0.8], respectively; Figure 2B-C).

The multimodal model similarly had the highest performance for 3-year EFS prediction with AUC: 0.88 (95% CI: 0.80–0.94), followed by DL-MRI alone AUC: 0.80 (95% CI: 0.69–0.91), and clinical features alone AUC: 0.69 (95% CI: 0.58–0.79) for the overall test set (Figure 2D). A similar pattern of stability across institutions was observed for 3-year EFS AUC as was observed for C-index (Figure 2E-F). Models that were trained only on one dataset (DF/BCH or CBTN) demonstrated degraded performance, though continued to show the benefit of a multimodal approach versus the use of clinical or DL-MRI features alone (Supplementary Material). Fine-tuning of a DF/BCH-trained model with samples of CBTN data was found to incrementally improve performance (Supplementary Material).

The time to predict EFS for each subject, from image processing to survival prediction, was approximately 2 minutes on a central processing unit with Intel(R) Xeon(R) Silver 4316, 2.30 GHz. Given the wide time range of diagnoses included, we evaluated the model’s performance by scan year and found that scan year had a negligible impact on model performance (Supplementary Material).

To evaluate the effect of transfer learning on model performance, we additionally trained a DL survival model to directly predict EFS from scratch without using the features coming from the pretrained segmentation model utilizing the T2-weighted MRI sequences. This approach yielded C-index: 0.64 (95% CI: 0.52–0.75) and AUC: 0.64 (95% CI: 0.58–0.69; Supplementary Figure S3) and poor stratification between low-risk group and high-risk group (Supplementary Figure S4).

### Model Calibration

**Figure 3.**
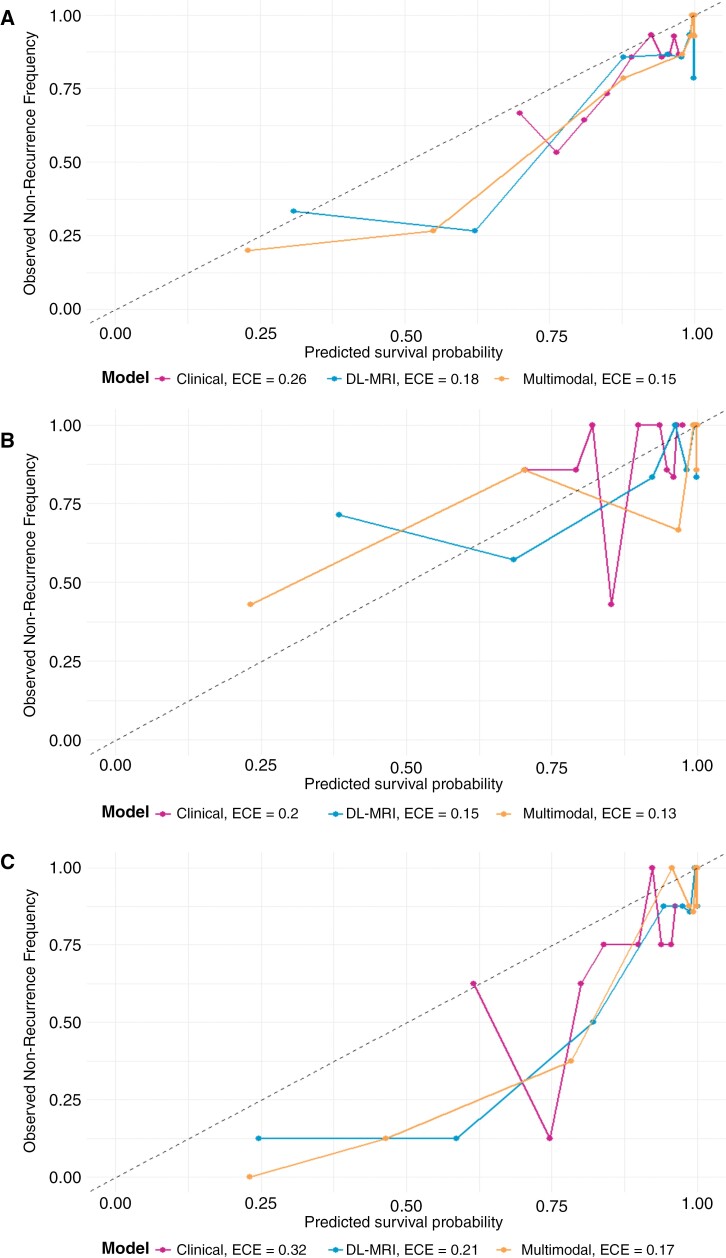
Calibration plots with 10 bins comparing model performance for test subjects: (A) total test, (B) DF/BCH cohort, and (C) CBTN cohort. A perfect prediction would follow the dashed diagonal line. expected calibration error (ECE) values are given for each model, indicating their predictive accuracy: lower ECE signifies closer alignment with the ideal line (best ECE equals zero).

Graphical calibration plots demonstrated adequate calibration across all 3 models, with slightly improved expected calibration error and Brier Scores for the multimodal model (Figure 3).

### Postoperative Risk Group Stratification With DL

**Figure 4.**
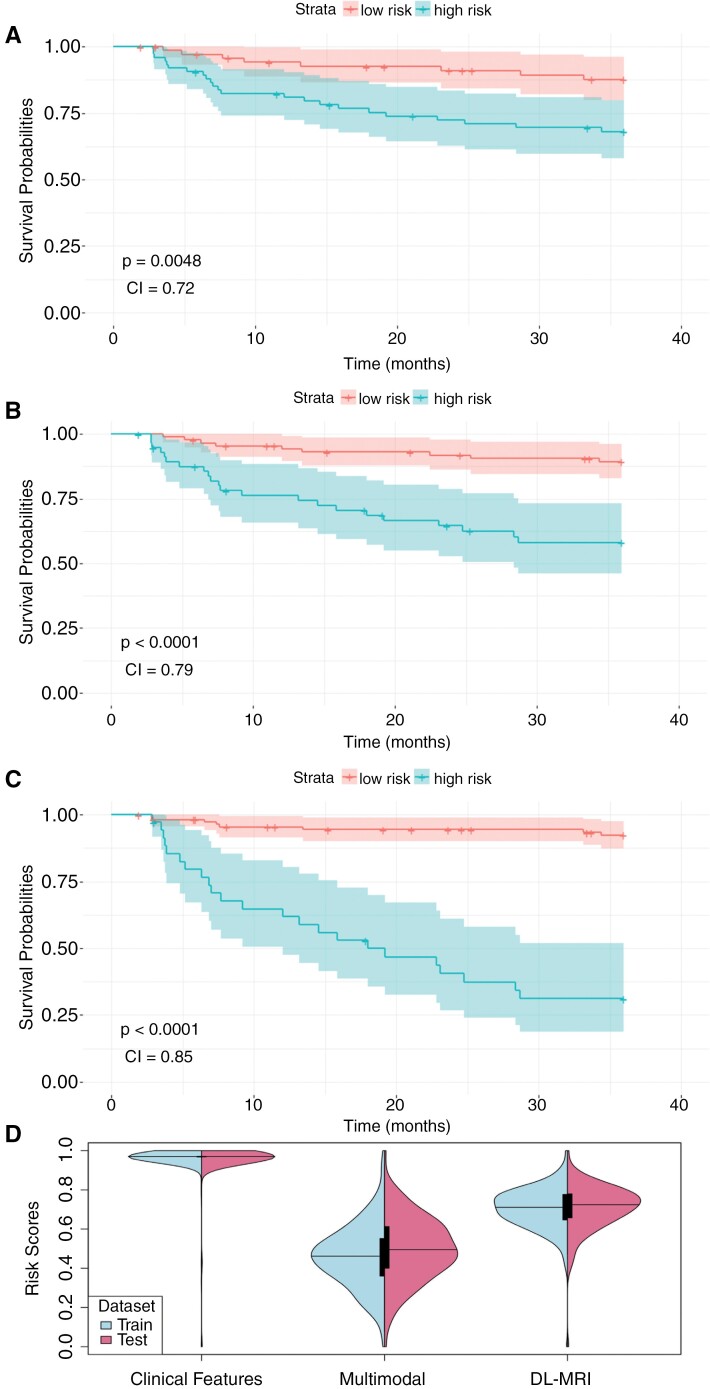
Kaplan–Meier survival curves stratifying patients into low and high-risk groups: (A) clinical feature model, (B) deep learning-MRI model, and (C) multimodal model. (D) Risk distributions for different models.

Patients in the test set were stratified into low and high-risk categories based on median risk scores from the holdout validation set (Supplementary Material). Kaplan–Meier survival plots by risk group indicate that the multimodal model yielded the most distinct, separable risk groups with a low-risk group with 3-year EFS 92% and high-risk group with 3-year EFS 31% (*P* < .0001; Figure 4). By comparison, the DL-MRI model yielded 3-year EFS of 89% and 58%, and the clinical feature model yielded 3-year EFS of 86% and 68% for low- and high-risk groups, respectively (Supplementary Figures S27-S32). To better understand the distribution of predicted EFS probabilities across the training and test sets, we generated violin plots for both training and testing sets across all 3 models, offering a visual and comparative analysis of the risk score dispersion. For the clinical model, the risk scores cluster towards the higher end, indicating a smaller absolute difference between low- and high-risk stratification, while the multimodal demonstrated a broader range of risk scores more closely aligned to true risk (Figure 4D).

### Clinical Examples of Improved Risk-Stratification With DL and Individual Survival Probability Curves

**Figure 5.**
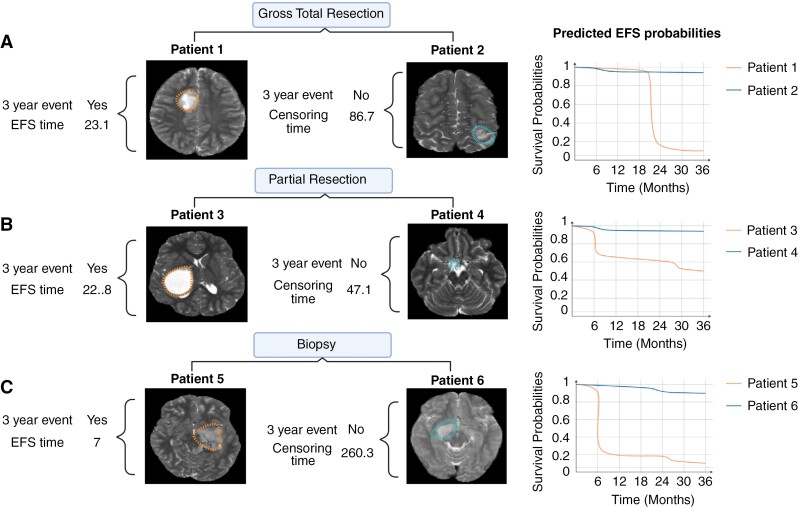
Correlation between magnetic resonance imaging (MRI) image features and 3-year event-free survival. MRI scans on the left display patients who eventually had recurrence within 3 years, and MRI scans on the right display patients without recurrence within the same period. Event-free survival curves for the corresponding patients are shown with the survival probabilities from the outputs of the multimodal model. (A) Patient 1 had a recurrence at 23.1 months with a survival probability of 0.2, while Patient 2 stayed recurrence-free with a 36-month survival probability of 0.9. (B) Patient 3 experienced a recurrence at 22.8 months with a survival probability of 0.6, compared to Patient 4, who remained free of recurrence with a survival probability of 0.9 at 36 months. (C) Patient 5 had a recurrence at 7 months with a survival probability of 0.3, whereas Patient 6 was recurrence-free with a 36-month survival probability of 0.9.

To demonstrate the potential utility of multimodal DL risk prediction, we investigated several example patient cases from the study test set, wherein baseline clinical risk factors were similar (ie, resection status), but patients had different recurrence patterns. In these cases, the incorporation of DL-MRI imaging features improved risk prediction for each patient (Figure 5). Furthermore, the logistic hazards model framework outputs sequential EFS probabilities over multiple time points, enabling the generation of a patient-specific survival probability curve, which may further inform patient management.^16^

## Discussion

In this study, we demonstrate that the incorporation of DL-based preoperative imaging features with clinical data can substantially improve postoperative recurrence risk prediction. A DL pipeline that leverages a routine preoperative MRI scan, age at diagnosis, and resection status could be a practical way of informing postoperative risk and, consequently, postoperative management decisions. For instance, identifying a patient as high-risk, which in the multimodal model, had 30% EFS at 3 years may prompt consideration of adjuvant therapy. In the era of investigative BRAF pathway-directed therapy, this model may also serve as a tool to select the ideal patients for clinical trials of adjuvant-targeted therapy. The study methods introduce a novel form of transfer learning—leveraging tumor segmentation knowledge—to overcome the limited data problem that has severely limited the application of DL to pediatric brain tumors. Our findings highlight the heterogeneous clinical trajectories of pLGGs across institutions and the limited prognostic capability of traditional clinical risk factors for this disease, including BRAF mutational status. By incorporating robust quantitative imaging features with key clinical factors, such as resection extent, DL may enable practical, accurate postoperative risk prediction for pLGG to support decision-making.

This is the first study, to the best of our knowledge, that examines multimodal DL for recurrence prediction in pLGG. Radiographic features of pLGGs have been well-documented in recent years.^2^ Certain morphological characteristics are associated with tumor grade and histology,^5^ though the association between particular features and risk of progression is unclear.^6^ More recently, several studies have investigated quantitative radiomics as a means to predict molecular subtype^45–47^ and progression risk^46,48^ for pLGGs. These early efforts have shown promising early results, though they have been limited by small datasets and the need for manually segmented tumor volumes, which are impractical to implement and lead to well-recognized problems with radiomic feature reproducibility.^45–48^ DL algorithms, particularly segmentation algorithms, by contrast, have been shown to be robust and reproducible in a number of settings.^20–22,30,49^ By utilizing a novel deep transfer learning approach with features derived from tumor segmentation, we obviate the need for manual segmentation and hypothesize that the approach will lead to predictions that are likely more robust than traditional radiomics for different patient and scanner characteristics.

Consistent with prior literature, we found that gross total resection was one of the strongest prognostic factors for pLGG.^50^ We additionally found that older age was protective, a finding that has been reproduced in some studies,^5,6^ but not others.^2,51^ Of note, BRAF mutational status was not found to be predictive of recurrence in our cohorts recognized in the literature as a factor associated with better. Some previous studies have linked BRAF mutation with poorer prognosis^52,53^ in pLGG, though others have found conflicting findings.^48^ We hypothesize that the effect of BRAF mutational status on recurrence is complex and may be dependent on the particular subtype of BRAF mutation, other tumor genomic factors, clinical factors, and radiomics.^48,54^

Our research also led to several intriguing technical discoveries. Firstly, it highlights the importance of pooling data to generate a more representative training cohort when dealing with a rare disease like pLGG that may present and be managed differently across institutions. We also find that fine-tuning smaller samples of external data, when possible, can also be an effective strategy to improve performance. Additionally, our use of a pretrained segmentation model, initially trained with subjects from the CBTN cohort, yielded consistent results across both DF/BCH and CBTN cohorts when used for MRI feature extraction in survival analysis. This consistency across diverse datasets underscores the effectiveness of the segmentation model in extracting meaningful image features that are informative across multiple institutions with varying scanner parameters. Lastly, we observed that models trained from scratch, as opposed to using a pretrained model for MRI feature extraction, demonstrated much lower performance and were nearly uninformative, indicating the utility of a transfer learning approach for DL classification on small datasets.

Our study has several limitations. The inherent heterogeneity of pLGG and the use of retrospective multi-institutional data may introduce selection biases and there were both known and likely unknown confounders. Despite the use of multi-institutional datasets and the largest study of its kind, the results necessitate external, independent validation to determine if the model is portable across other centers managing pLGG. We found that model performance degraded a bit when applied more generally. Specifically, when the model was trained with data from one site and applied to data from another site, performance degraded, indicating that larger, diverse multicenter training data is likely necessary to build a generalizable model that performs stably out-of-the-box, without the need for fine-tuning. The role of adjuvant therapy in pLGG is controversial and may vary by institutional preferences. Only a small proportion of patients in our datasets received adjuvant therapy. While neither radiation nor chemotherapy was associated with EFS, we were unable to fully investigate the interaction between the model performance and adjuvant therapy use, and this warrants further investigation. We hope that the public release of the source code^55^ and the model will facilitate further validation and clinical translation of our work.

## Conclusion

We developed a multimodal DL framework using preoperative MRI and several EHR-derived clinical variables to improve postoperative recurrence prediction and risk stratification for pediatric low-grade gliomas. DL can yield informative MRI-extracted features to substantially improve risk prediction beyond the current standard. We publish a practical and hands-free model to encourage clinical translation. External validation and prospective study of this model is needed to determine how it can improve personalized postoperative management and ultimately improve patient outcomes.

## Supplementary material

Supplementary material is available online at *Neuro-Oncology* (https://academic.oup.com/neuro-oncology).

noae173_suppl_Supplementary_Materials

## Data Availability

Raw MRI imaging data cannot be shared at this time due to hospital use agreements and patient privacy concerns, but for replicating the analysis the code is shared.

## Code availability

The code is publicly available.^55^
