# Supplementary material for: Multimodal deep learning improves recurrence risk prediction in pediatric low-grade gliomas
Source: Neuro Oncol. 2024 Aug 30;27(1):277–90. doi: 10.1093/neuonc/noae173 (PMC11726244; doi:10.1093/neuonc/noae173)
Supplement: noae173_suppl_Supplementary_Materials [file noae173_suppl_supplementary_materials.docx]

*Supplementary information for:*

**Multimodal Deep Learning Improves Recurrence Risk Prediction in Pediatric Low-Grade Gliomas**

**Table of Contents**

[List of abbreviations 2](#_Toc169250590)

[Supplementary Methods 3](#_Toc169250591)

[A.1 Subject selection workflow 3](#_Toc169250592)

[A.2 Survival model details 4](#_Toc169250593)

[A.3 Kaplan-Meier curves across various survival models in the training set 5](#_Toc169250594)

[A.4 Survival model trained from scratch 6](#_Toc169250595)

[A.5 Clinical variable selection 8](#_Toc169250596)

[A.6 Impact of BRAF mutation on EFS prediction 12](#_Toc169250597)

[A.7 Evaluation metrics 14](#_Toc169250598)

[A.8 Comparison of early and later MR imaging of DF/BCH cohort 15](#_Toc169250599)

[A.9 Model performance with separate DF/BCH and CBTN training and testing 17](#_Toc169250600)

[A.10 Impact of fine-tuning on risk prediction 22](#_Toc169250601)

[Supplementary Figures 27](#_Toc169250602)

[Supplementary Tables 32](#_Toc169250603)

[References 34](#_Toc169250604)

# List of abbreviations

pLGG – pediatric low-grade glioma

EFS – event-free survival

DF/BCH – Dana Farber/Boston children’s hospital

CBTN – children’s brain tumor network

DL – deep learning

CI – C Index

AUC – area under the curve

GTR – gross total resection

STR – subtotal resection

NF1 - neurofibromatosis type 1

ReLU - Rectified Linear Unit

# Supplementary Methods

## A.1 Subject selection workflow

The DF/BCH and CBTN cohorts initially comprised 303 and 524 subjects, respectively. The inclusion criteria for the study were stringent, with Figure S1 illustrating the process. Eligible subjects required T2-weighted MR scans, a diagnosis of low-grade glioma, and preoperative MR scans. Surgical intervention was a prerequisite, with biopsies also qualifying as surgical procedures. Subjects with NF1 were excluded due to their distinct prognosis, as were those with metastatic disease, which impacts outcomes differently compared to non-metastatic cases. Additionally, accurate EFS predictions necessitated the exclusion of subjects without reported event times.
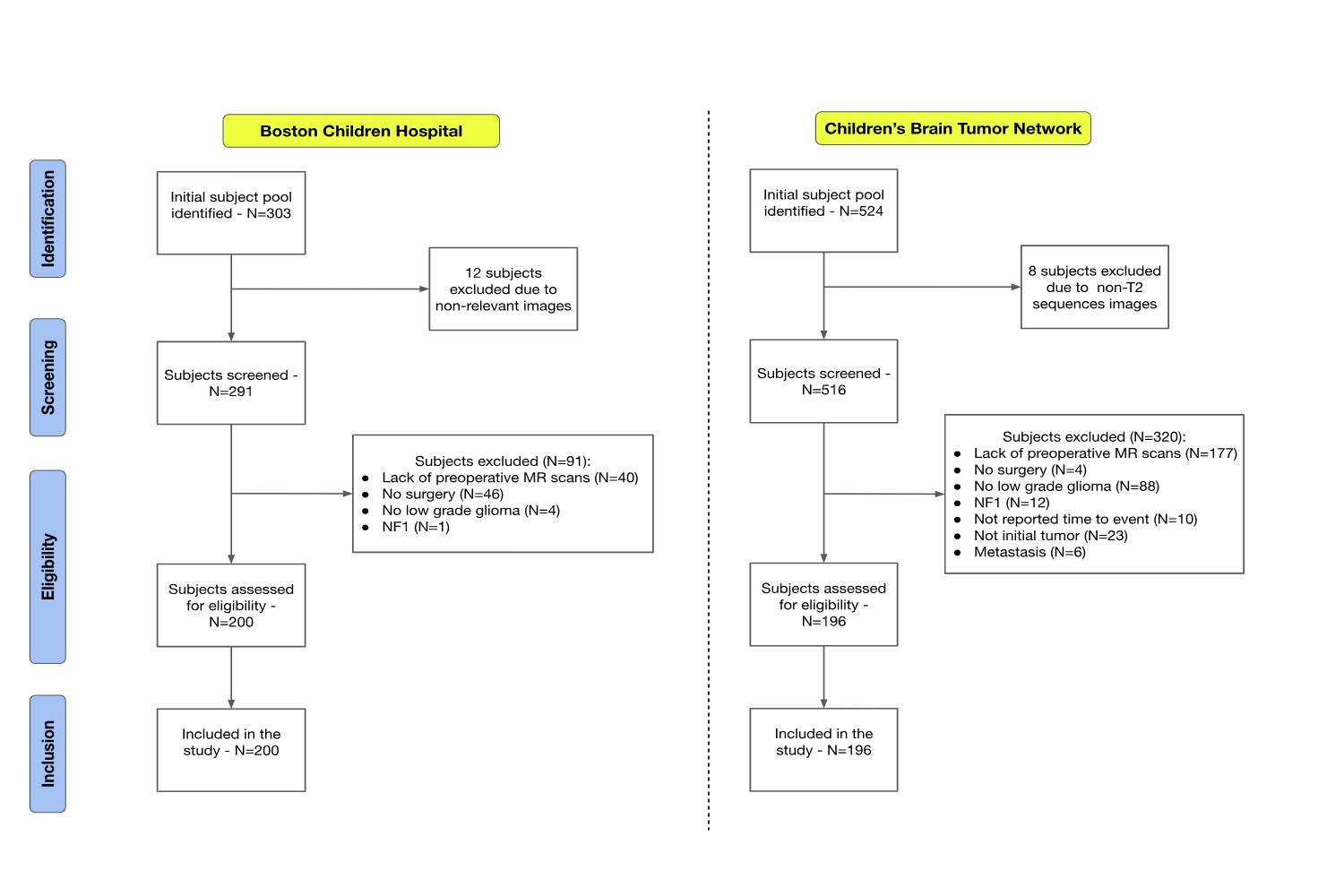
 Figure S1. This figure outlines the stages from identification to inclusion, detailing the initial number of subjects, screening exclusions, eligibility assessments, and the final count of subjects incorporated into the study.

## A.2 Survival model details

The feature vectors were normalized using the mean and standard deviation derived from the training dataset, to ensure consistency and comparability across the dataset, then they are fed to the survival network. The survival network has three layers and neuron counts of 2000, 200, and 15, uses dropout rate (0.3) and batch normalization to avoid overfitting, with ReLU activation for non-linearity. Originally set for 15-time intervals, the model uses linear interpolation to expand to 500 intervals, improving the detail and accuracy of survival predictions. Models were constructed and trained using Pycox and torch. The survival network was trained over 100 epochs with early stopping, halting if the loss didn't decrease for 10 epochs to prevent overfitting. It used the Adam optimizer for refining parameters and minimizing prediction errors.

It employed a specialized logistic hazards loss function for survival model training, given by:

$$L = - \sum_{\left\{ i=1 \right\}}^{n} \sum_{\left\{ t=1 \right\}}^{T} \left( \delta_{\left\{ i,t \right\}}\cdot\log\left( p_{\left\{ i,t \right\}} \right)+ \left( 1 - \delta_{\left\{ i,t \right\}} \right)\cdot\log\left( 1 - p_{\left\{ i,t \right\}} \right) \right),$$

where n is the number of individuals in the dataset. T is the number of discrete time intervals. δ_i,t_ is an indicator function that equals 1 if the event for individual i occurs at time t, and 0 otherwise. p_i,t_ is the predicted probability of the event occurring at time t, given it has not occurred before, for individual i.

In our study, the dataset was partitioned into two primary subsets: 70% for development purposes and 30% for testing. The development set underwent a three-fold cross-validation process to evaluate the model's mean survival performance. Prior to initiating cross-validation, one step was taken to reserve 10% of the development data as a validation holdout. This decision was made to align the validation process with the requirements of constructing Kaplan-Meier curves. The Kaplan-Meier survival analysis needs to divide patients into low- and high-risk groups. To ensure consistency and reliability in this division, we opted to utilize the mean risk score derived from the validation holdout as a benchmark. This approach not only facilitated a standardized comparison between the predicted risks and the actual outcomes but also played a role in the training phase. The validation holdout was instrumental in implementing early stopping criteria during model training, thereby preventing overfitting and ensuring that the model generalizes well to unseen data. By integrating the validation holdout in both the training optimization and the survival analysis framework, we aimed to enhance the model's predictive accuracy and validity in estimating patient survival probabilities.

## A.3 Kaplan-Meier curves across various survival models in the training set

Panel A of Figure S2 displays Kaplan-Meier curves for the training cohort using the clinical model. The curves demonstrate a statistically significant distinction between the survival of low and high-risk groups, with the high-risk group showing a 3-year EFS rate of 0.71 compared to 0.90 for the low-risk group. Panel B focuses on survival curves derived from DL-MRI image features. This model exhibits superior risk stratification, as evidenced by a lower p-value and a higher C-index relative to the clinical model in Panel A. The 3-year EFS rates are markedly different, at 0.49 for the high-risk and 0.94 for the low-risk group. Panel C presents Kaplan-Meier curves using a model that integrates both clinical variables and DL-MRI image features. This combined model achieves the highest C-index amongst the training set models and shows significant discrimination between low and high-risk groups, with respective 3-year EFS rates of 0.93 and 0.20.


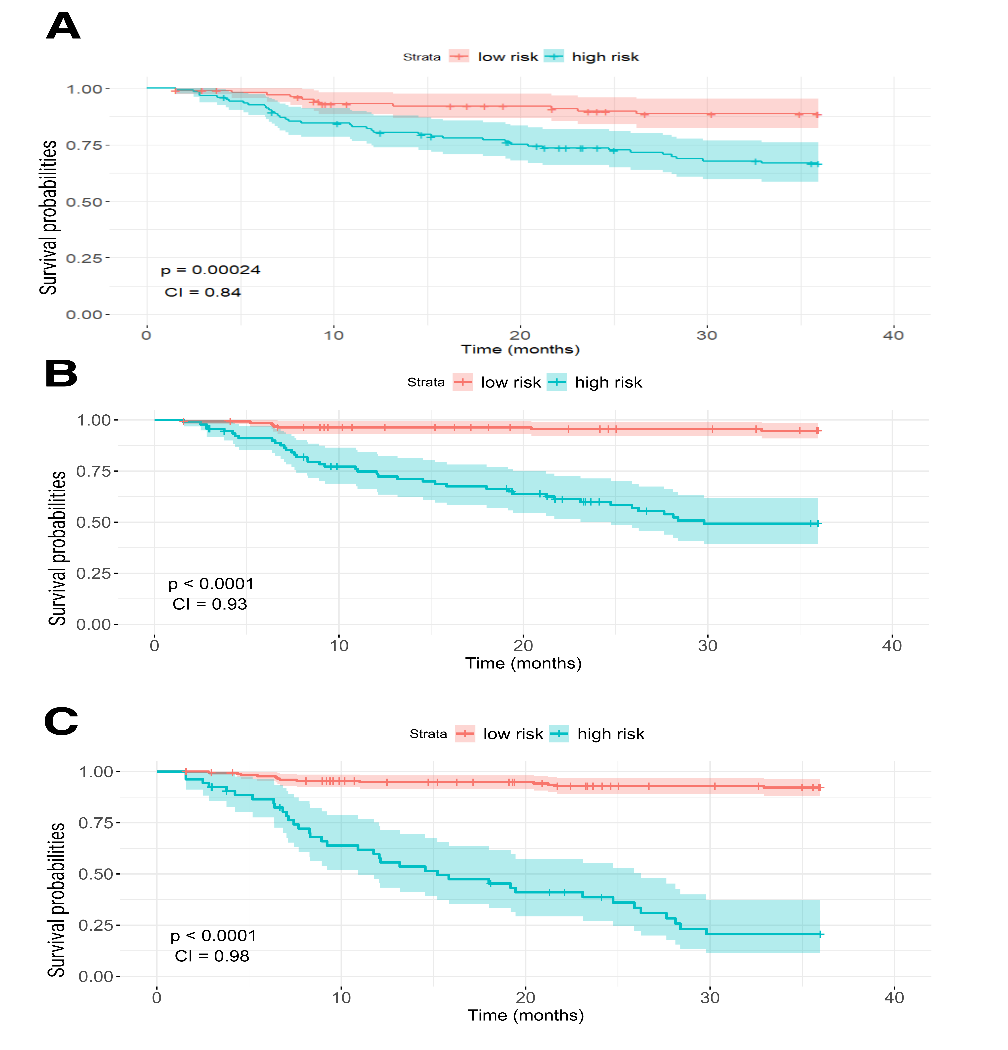


Figure S2. Kaplan-Meier survival plots generated for the training set based on the output risk scores of different developed survival models. (A) clinical model, (B) DL-MRI model, and (C) multimodal.

## A.4 Survival model trained from scratch

We embarked on developing a model, employing convolutional layers, to train pediatric event-free survival from scratch. This approach was distinct from using a pretrained segmentation model. Our objective was to compare the performance metrics of two models: one that extracts image features from scratch and another that relies on features derived from a pretrained pediatric segmentation model.

For this purpose, we selected HighResNet^1^ as the foundation for our from-scratch training, recognizing its proficiency in capturing image features. After flattening these features for the survival layers, we adopted the same architectural design used in our survival model based on pretrained segmentation. Also, we used the same training, validation and test dataset for gaining the result. This was done to ensure a fair comparison between the two models.

Our comparative analysis focused on the Image model versus this new scratch-based model, specifically excluding the model based on both clinical variables and images from this evaluation. The aim was to distinctly assess the impact of image features extracted from a scratch-built model against those derived from a pretrained segmentation model on the performance of the survival model.

The analytical results, comprising performance metrics and Kaplan-Meier curves for both low and high-risk groups, suggest that the model developed from scratch yielded a low AUC, indicating a limited ability to effectively distinguish between the low and high-risk groups.

Our attempts to train the model anew, foregoing the use of pretrained segmentation for feature extraction, did not yield satisfactory accuracy levels. Figure S3 illustrates the ROC curves for the DF/BCH cohort, the CBTN cohort, and the entire test set. The AUC metrics observed do not reach the threshold necessary to indicate a robust prediction model.


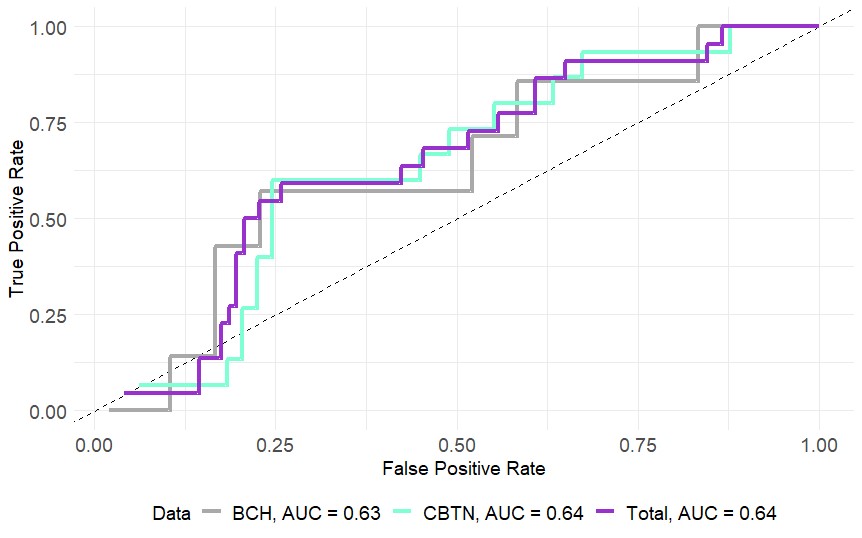


Figure S3. ROC curves representing various subgroups within the test cohort, derived from a model trained from scratch with MR images as the input.

Figure S4 presents the Kaplan-Meier survival curves for the test set, highlighting the differentiation between the low-risk and high-risk groups. The low AUC, low C-index, and high p-value (greater than 0.05) observed indicate a lack of effective discrimination by the model between the two risk categories.


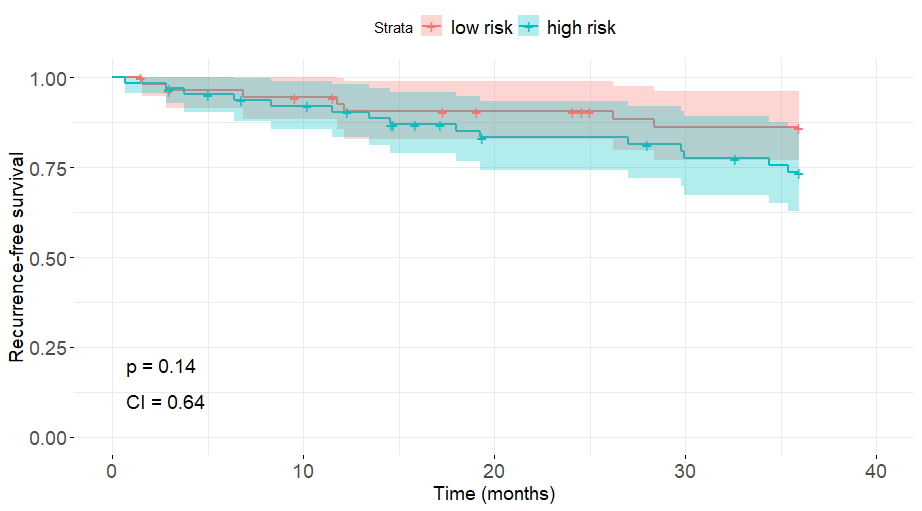


Figure S4. Kaplan-Meier survival curves were generated for the whole test set using a model trained from scratch with MR images as the input.

## A.5 Clinical variable selection

For choosing clinical variables for predicting recurrence outcomes, we began with a set of existing clinical variables common to both cohorts, which included age, gender, resection status, chemotherapy, radiotherapy, location of tumor, and BRAF mutation. To determine the relevance of each variable for predicting survival outcomes, we employed univariable survival models. Our primary tools for evaluation were the Bayesian Information Criterion (BIC) and p-value analysis. The BIC was particularly useful in assessing the balance between model simplicity and fit; a lower BIC indicated a preferable model^5^. In our approach to selecting clinical variables, we initially focused on those with the lowest p-value and BIC, gradually incorporating additional variables. This methodical process was guided by the Cox proportional hazards model^6^, ensuring an effective balance between model accuracy and complexity.

Table S1 presents hazard ratios and their 95% confidence intervals from both univariable and multivariable models, along with the p-values indicating the significance of each variable in predicting outcomes. We designated a p-value of < 0.15 as significant.

Table S1. Univariable and multivariable predictors of EFS.

| Variable | Univariable Analysis | | Multivariable Analysis | |
| --- | --- | --- | --- | --- |
|  | HR (95% CI) | P Value | HR (95% CI) | P Value |
| Age | | | | |
| Continuous, yearly | 0.999918 (0.999807-1.000028) | 0.144625 | 0.999899 (0.999788-1.000010) | 0.07565549 |
| Gender | | | | |
| Female | [Reference] | | | |
| Male | 1.036949 (0.686128-1.567147) | 0.863284 | 1.038696 (0.683258-1.579038) | 0.8589914 |
| Resection Status | | | | |
| GTR | [Reference] | | | |
| Biopsy | 3.508367 (1.941205-6.340722) | < 0. 001 | 3.312682 (1.820156-6.029078) | < 0. 001 |
| STR | 4.074138 (2.473604-6.710291) | < 0. 001 | 4.208365 (2.548813-6.948465) | < 0. 001 |
| Chemotherapy | | | | |
| Yes | [Reference] | | | |
| No | 2.236749 (0.311428-16.064872) | 0.423556 | 3.856904 (0.444491-33.466856) | 0.2207776 |
| Radiotherapy | | | | |
| Yes | [Reference] | | | |
| No | 1.233645 (0.171873-8.854686) | 0.834604 | 0.516094 (0.060555-4.398527) | 0.5451493 |
| BRAF | | | | |
| V600E | [Reference] | | | |
| Fusion | 0.889032 (0.504868-1.565513) | 0.683697 | 0.847754 (0.473980-1.516283) | 0.5776900 |
| Wild | 1.127979 (0.660833-1.925352) | 0.658890 | 1.180793 (0.689471-2.022234) | 0.5449083 |
| Tumor Location | | | | |
| Supratentorial | [Reference] | | | |
| Non-Cortical Supratentorial | 1.518422 (0.833170-2.767268) | 0.172587 | 1.005807 (0.539367-1.875620) | 0.9854712 |
| OpticPathway/Non-Cortical Supratentorial | 2.096836 (0.965881-4.552033) | 0.165931 | 1.176212 (0.530186-2.609414) | 0.6897390 |
| Posterior Fossa | 0.615444 (0.352000-1.076055) | 0.187423 | 0.754786 (0.425675-1.338347) | 0.3357099 |
| Brainstem | 1.552414 (0.782498-3.079868) | 0.208292 | 1.044629 (0.513574-2.124816) | 0.9040668 |
| Spinal Cord | 1.231922 (0.376987-4.025685) | 0.729916 | 0.699842 (0.194508-2.518031) | 0.5848368 |
| Other | 1.357702 (0.185502-9.937118) | 0.763335 | 0.687436 (0.091001-5.193004) | 0.7164028 |

Due to the absence of BRAF labels for all patients, we employed a model to derive these labels. We then excluded patients without BRAF data to conduct the univariable analysis with only those having labeled BRAF mutations which is addressed in Table S2. This adjustment slightly altered the p-values, but the results still indicated no significant difference in outcomes among different BRAF mutations.

Table S2. Univariable analysis of EFS predictors for BRAF mutations, excluding subjects with inferred BRAF mutations.

| Variable | Univariable Analysis | |
| --- | --- | --- |
|  | HR (95% CI) | P Value |
| V600E | Reference | |
| Fusion | 1.122014 (0.515277-2.443179) | 0.373616 |
| Wild | 1.196228 (0.520096-2.751343) | 0.570672 |

## A.6 Impact of BRAF mutation on EFS prediction

Given the absence of BRAF mutation data for all subjects, we applied a previously validated model to estimate mutations for those lacking explicit data. Our examination of the BRAF mutation's impact on survival prediction within the DF/BCH cohort revealed that 150 out of 200 patients did not contain the BRAF mutation variable, while in the CBTN cohort, 101 out of 196 were without the mutation. To assess the mutation's presence, we utilized a classifier model developed by Tak et al^2^. This model discriminates among V600E, fusion, and wild-type BRAF mutations in MRI images with notable accuracy. To determine the significance of the BRAF mutation in recurrence, we conducted two analyses: one excluding the predicted BRAF mutation data to ascertain the variance in survival among the groups, and another including it. Contrary to existing literature that suggests poorer survival rates in pediatric low-grade glioma patients with the BRAF V600E mutation^3,4^, our findings did not show a clear distinction in overall recurrence or three-year recurrence outcomes between V600E, fusion, and wild-type mutations in both analyses.

To verify whether outcomes differ among patients with three types of BRAF mutations - V600E, fusion, and wild-type - we generated Kaplan-Meier curves for each category using our dataset. Figure S5 presents these curves for the specific categories of BRAF mutations, yielding a p-value of 0.9 when considering subjects with confirmed BRAF mutation data, using V600E as the reference. Meanwhile, Figure S6 shows the Kaplan-Meier curves with a p-value of 0.4, which includes subjects with both confirmed and estimated BRAF mutation data, again using V600E as the reference point.


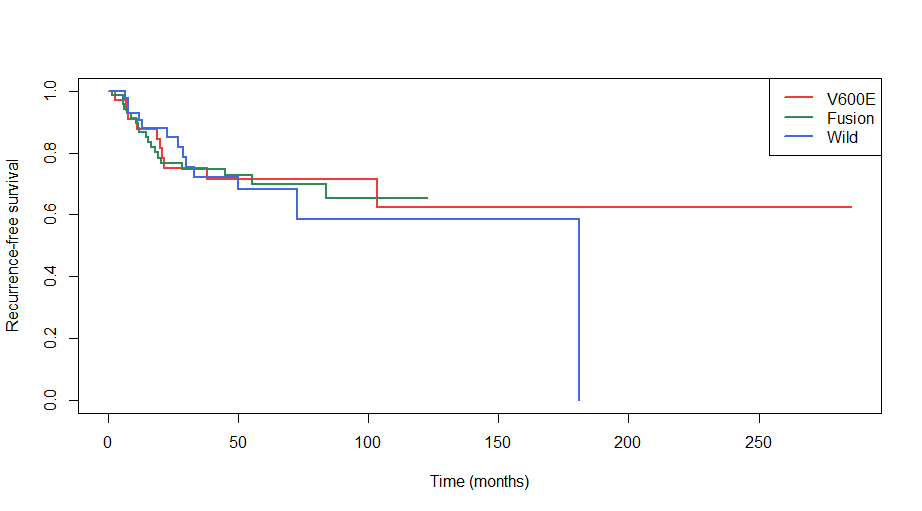


Figure S5. Variations in survival outcomes across distinct BRAF mutation statuses, excluding cases with predicted BRAF mutation labels.


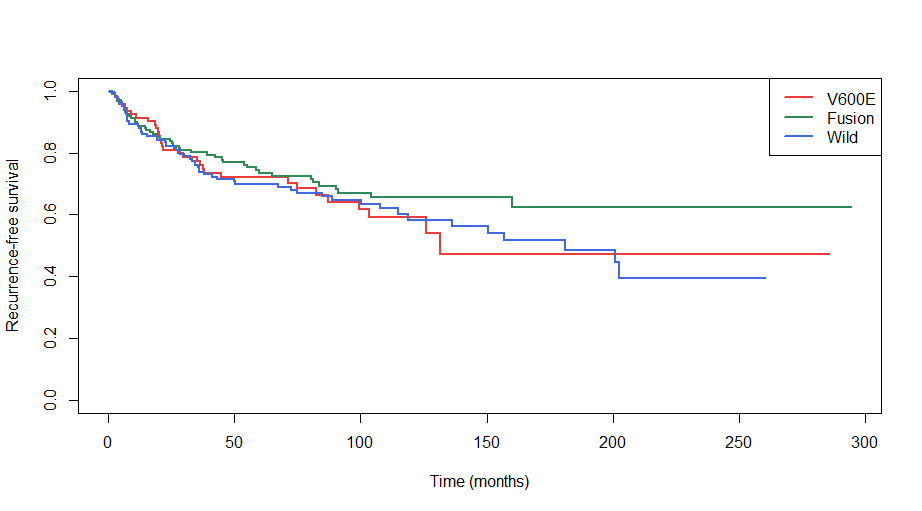


Figure S6. Variations in survival outcomes across distinct BRAF mutation statuses, including cases with predicted BRAF mutation labels.

Furthermore, we attempted to develop a hybrid EFS model incorporating clinical variables and MRI features. The clinical variables included BRAF mutation status, age, and resection status. Figure S7 depicts the ROC curves for this model across the test cohort. Judging by the AUC values, it appears that this EFS model performs inferiorly compared to the one solely based on MRI features, suggesting that integrating BRAF mutation as a clinical variable misleads the EFS model.


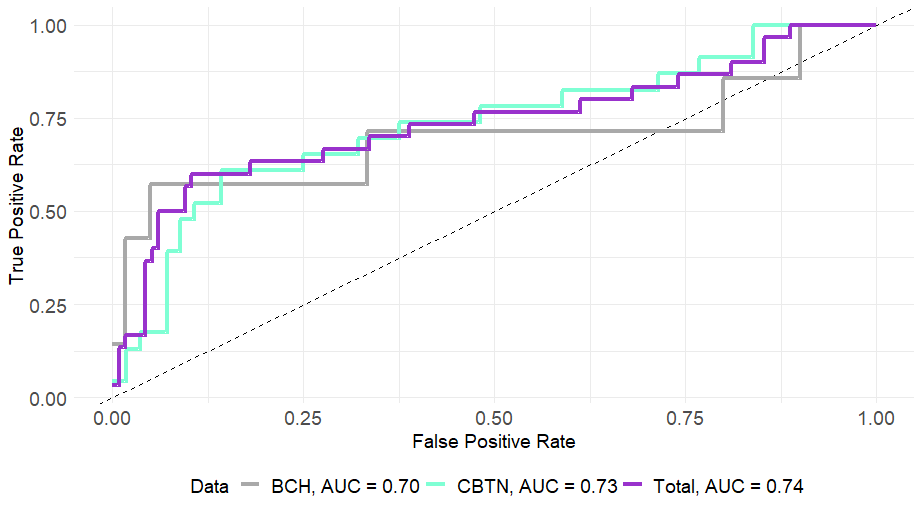


Figure S7. ROC curves representing various subgroups within the test cohort, derived from a model with MR images, BRAF, age, and resection status as the input.

## A.7 Evaluation metrics

C Index: The C-index measures the agreement between predicted risks and actual event times, considering only pairs where one event precedes another. Concordance occurs when higher risk predictions align with shorter event times. It ranges from 0 to 1, with 0.5 denoting no prediction ability and 1 indicating perfect prediction^10^.

AUC: The AUC (Area Under the Curve) was obtained by plotting the true positive rate (sensitivity) against the false positive rate (1 - specificity) and provides a single measure of the model's ability to distinguish between those who experienced the recurrence and those who did not. An AUC of 1 indicates perfect discrimination, while an AUC of 0.5 indicates a lack of discriminative capacity^7^.

Calibration: A calibration plot is a graphical representation that compares the predicted probabilities of an event from a statistical model against the observed frequencies of the event, used to assess the accuracy of the model's predictions^9^. Each point on these plots corresponds to a specific bin of predicted probabilities, with its mean predicted probability on the x-axis and the actual observed frequency of non-recurrence events on the y-axis. The closer these points are to the dashed diagonal line, which represents perfect calibration, the more accurate the model is in its predictions.

IBS: The Integrated Brier Score (IBS) is a robust metric for the assessment of survival predictions, which also correlates with the calibration plot. The IBS measures the average squared difference between the observed event times and the predicted survival probabilities over time. The optimal IBS is zero, and lower values signify greater predictive accuracy of the model^9^. The results showed that the clinical features model had an IBS of 0.12, the DL-MRI model recorded an IBS of 0.09, and the multimodal model achieved the best performance with an IBS of 0.07.

## A.8 Comparison of early and later MR imaging of DF/BCH cohort

Given the wide range of the DF/BCH cohort from 1992 to 2019, we decided to compare the image quality and potential bias between early and later MR imaging. We divided the DF/BCH dataset into five bins based on the scan date years. The distribution of the number of patients in each year bin is illustrated in the following plot.


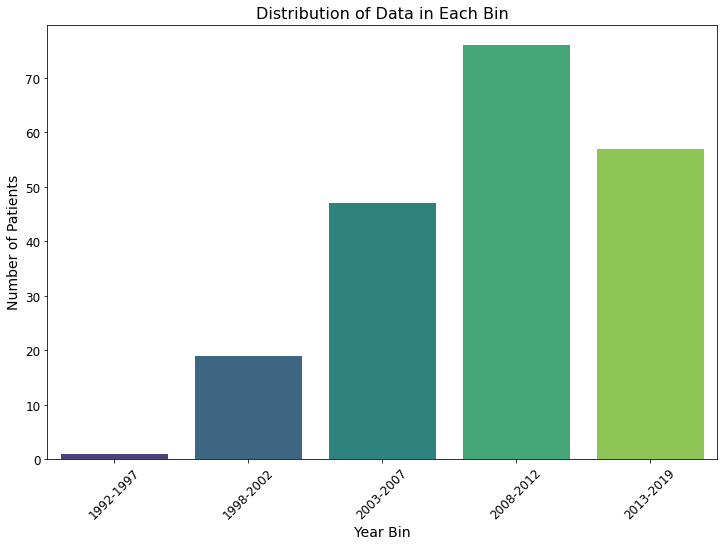


Figure S8. Distribution of patients in DF/BCH based on the year of scan date.

We compared the scan qualities based on resolution and slice thickness. For resolution, we examined the voxel sizes in the x and y dimensions, while for slice thickness, we considered the z dimension. We calculated the average values of the x, y, and z dimensions for each year bin. The following plots illustrate the changes in the x, y, and z dimensions over time.


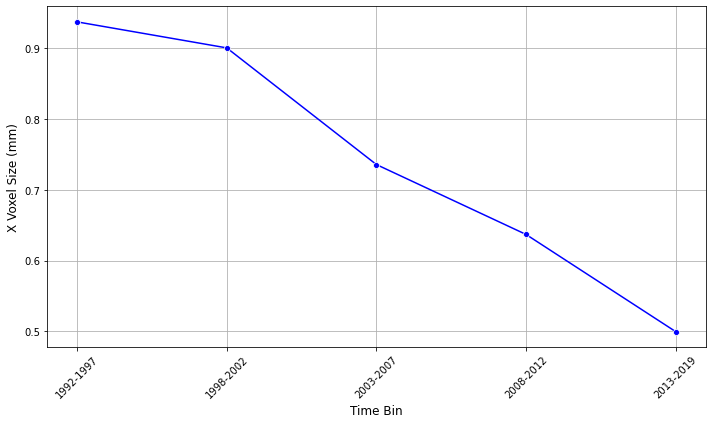


Figure S9. Average X voxel size in the DF/BCH cohort, categorized by scan date year.


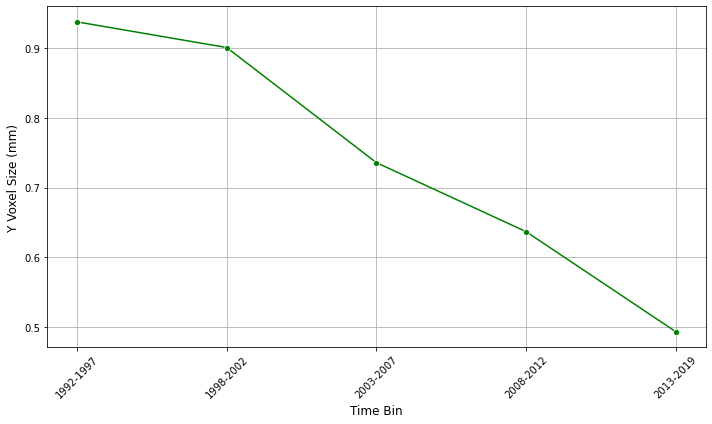


Figure S10. Average Y voxel size in the DF/BCH cohort, categorized by scan date year.


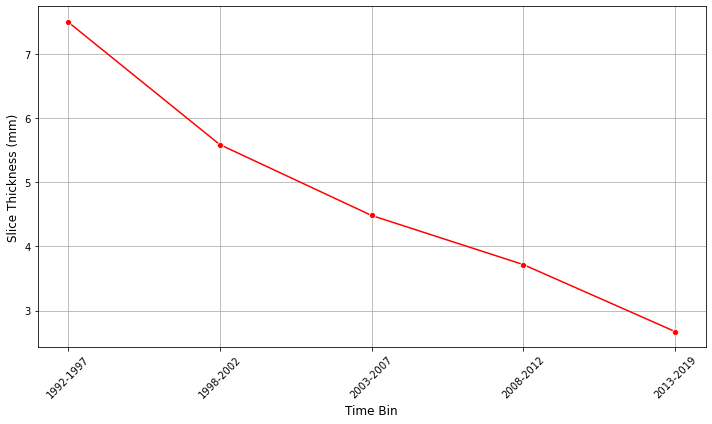


Figure S11. Average Z voxel size in the DF/BCH cohort, categorized by scan date year.

A decrease in the voxel sizes of the x and y dimensions indicates an increase in image resolution and image quality. Similarly, a decrease in the z dimension signifies a reduction in slice thickness, which corresponds to an improvement in image quality. As the years progress, we observe an improvement in image quality. This trend is expected, as advancements in MRI technology have led to better resolution and thinner slices, resulting in higher quality images.

Then to understand if newer, higher-quality images yield better results, we divided the test set of the DF/BCH dataset into five bins based on the year of the scan date. This allowed us to compare performance metrics across different time periods. We conducted experiments using only the model based on images, excluding clinical data, to focus specifically on the impact of image quality. In the test set containing DF/BCH subjects, we had 0 subjects in bin 1, 11 in bin 2, 11 in bin 3, 23 in bin 4, and 21 in bin 5. The C-indexes for bins 2 to 5 were 1, 0.7, 0.82, and 0.78, respectively. The corresponding AUCs were 1, 0.7, 0.81, and 0.72, and the IBS numbers were 0.02, 0.13, 0.11, and 0.08. When comparing these metrics across the year bins, we did not observe a consistent improvement in C-index and AUC performance metrics, nor a consistent decrease in IBS, despite the improved image qualities in later years. This indicates that better image quality does not necessarily translate to improved model performance.

## A.9 Model performance with separate DF/BCH and CBTN training and testing

To investigate the generalizability of learned model features from one institution to another, we trained two separate models; one trained using DF/BCH data (n=140) and tested on the CBTN validation set (n=59), and one trained using CBTN data (n=137) and tested on the DF/BCH validation set (n=60). For each model, we generated three versions, matching experiments from our pooled model: clinical features-only, DL-MRI features only, and multimodal clinical and imaging features. The validation sets used were fixed from our prior pooled experiments to enable direct performance comparisons. The models were developed for predicting three-year recurrence, and the following C-index and AUC values pertain to three-year EFS. The results are as follows:

**DF/BCH–Trained Model**

1. Clinical Model:

- C-index:
  - Train >> DF/BCH: 0.87 (95% CI: [0.78, 0.94])
  - Test >> CBTN: 0.52 (95% CI: [0.39, 0.67])
- AUC:
  - Train >> DF/BCH: 0.64 (95% CI: [0.45, 0.81])
  - Test >> CBTN: 0.40 (95% CI: [0.26, 0.56])


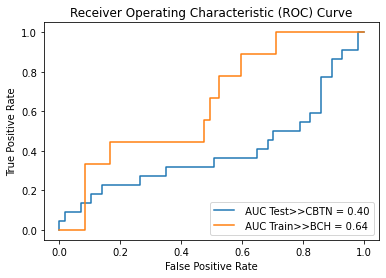


Figure S12. AUC curves for the clinical model trained on DF/BCH and tested on CBTN.

1. DL-MRI Model:

- C-index:
  - Train >> DF/BCH: 0.92 (95% CI: [0.85, 0.98])
  - Test >> CBTN: 0.52 (95% CI: [0.39, 0.64])
- AUC:
  - Train >> DF/BCH: 0.92 (95% CI: [0.82, 0.99])
  - Test >> CBTN: 0.55 (95% CI: [0.39, 0.69])


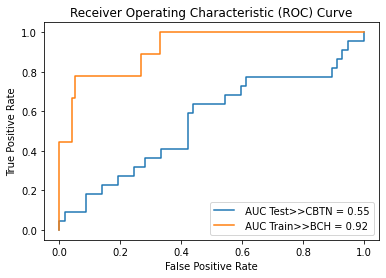


Figure S13. AUC curves for the DL-MRI model trained on DF/BCH and tested on CBTN.

1. Multimodal Model:

- C-index:
  - Train >> DF/BCH: 0.91 (95% CI: [0.84, 0.98])
  - Test >> CBTN: 0.54 (95% CI: [0.4, 0.66])
- AUC:
  - Train >> DF/BCH: 0.85 (95% CI: [0.69, 0.97])
  - Test >> CBTN: 0.62 (95% CI: [0.48, 0.75])


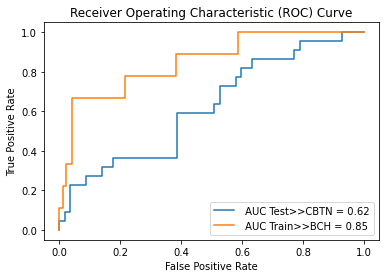


Figure S14. AUC curves for the Multimodal model trained on DF/BCH and tested on CBTN.

**CBTN-Trained Model:**

1. Clinical Model:

- C-index:
  - Train >> CBTN: 0.75 (95% CI: [0.63, 0.8])
  - Test >> DF/BCH: 0.59 (95% CI: [0.41, 0.78])
- AUC:
  - Train >> CBTN: 0.74 (95% CI: [0.63, 0.84])
  - Test >> DF/BCH: 0.49 (95% CI: [0.33, 0.66])


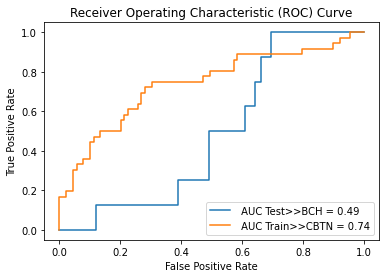


Figure S15. AUC curves for the clinical model trained on CBTN and tested on DF/BCH.

1. DL-MRI Model:

- C-index:
  - Train >> CBTN: 0.93 (95% CI: [0.89, 0.97])
  - Test >> DF/BCH: 0.57 (95% CI: [0.40, 0.78])
- AUC:
  - Train >> CBTN: 0.87 (95% CI: [0.79, 0.94])
  - Test >> DF/BCH: 0.56 (95% CI: [0.35, 0.76])


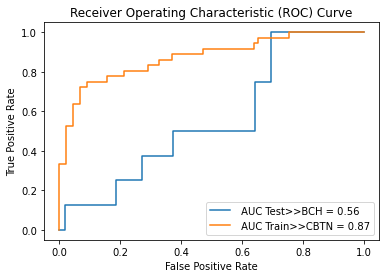


Figure S16. AUC curves for the DL-MRI model trained on CBTN and tested on DF/BCH.

1. Multimodal Model:

- C-index:
  - Train >> CBTN: 0.93 (95% CI: [0.90, 0.97])
  - Test >> DF/BCH: 0.66 (95% CI: [0.51, 0.81])
- AUC:
  - Train >> CBTN: 0.97 (95% CI: [0.9, 0.99])
  - Test >> DF/BCH: 0.62 (95% CI: [0.37, 0.85])


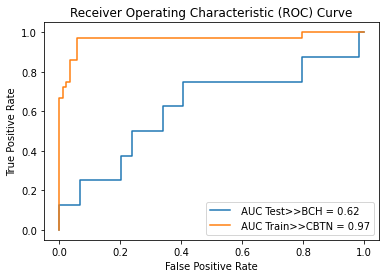


Figure S17. AUC curves for the Multimodal model trained on CBTN and tested on DF/BCH.

## A.10 Impact of fine-tuning on risk prediction

Given degradation in performance of the models trained on only one dataset, we investigated incremental fine-tuning on external data to determine how increasing data availability would improve model performance at an external setting. We specifically utilized the DF/DF/BCH-trained model as a baseline, and incrementally incorporated CBTN data into the model training, in increments of 20% (stratified by event presence), preserving the original CBTN hold-out validation set for performance evaluation.

Experiment 1: 20% of CBTN data was added to the DF/BCH training set.

Experiment 2: 40% of CBTN data was added to the DF/BCH training set.

Experiment 3: 60% of CBTN data was added to the DF/BCH training set.

Experiment 4: 80% of CBTN data was added to the DF/BCH training set.

The following table details information about the experiments. The model with 100% of CBTN training data available for fine-tuning is equivalent to the pooled model presented as our primary model in the manuscript.

Table S3. Number of subjects and training-to-test ratio for each incremental fine-tuning experiment.

| Percent of the CBTN training subjects available for fine-tuning | 0% | 20% | 40% | 60% | 80% | 100% |
| --- | --- | --- | --- | --- | --- | --- |
| Number of CBTN subjects in Training | 0 | 24 | 49 | 73 | 97 | 122 |

The AUC plots for experiments 1 through 4 are provided below.


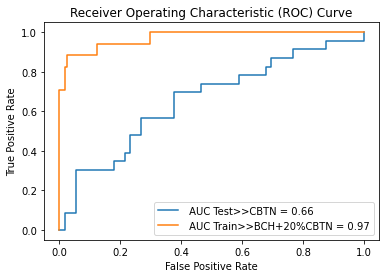


Figure S18. AUC of the multimodal survival model on the blinded CBTN test set and the training set containing the remaining 20% of CBTN data.


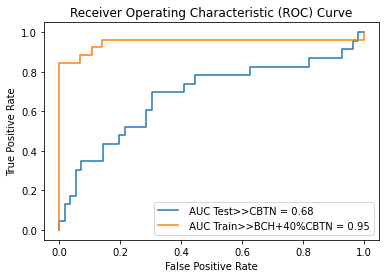


Figure S19. AUC of the multimodal survival model on the blinded CBTN test set and the training set containing the remaining 40% of CBTN data.


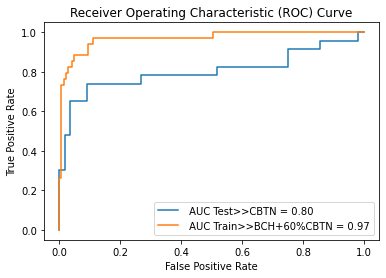


Figure S20. AUC of the multimodal survival model on the blinded CBTN test set and the training set containing the remaining 60% of CBTN data.


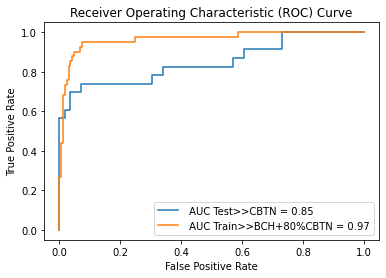


Figure S21. AUC of the multimodal survival model on the blinded CBTN test set and the training set containing the remaining 80% of CBTN data.

In the following figures, we present the C-index and AUC plots for the multimodal survival model. These plots are based on the same blinded CBTN test set, while the model was trained using different percentages of CBTN data added to the DF/BCH training set.


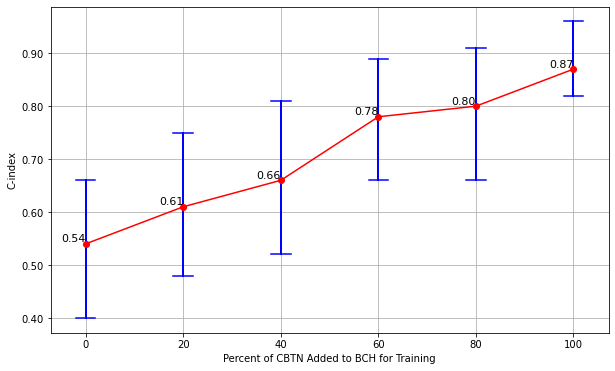


Figure S22. C-index with 95% confidence intervals for the multimodal survival model evaluated on the blinded CBTN test set with incrementally increasing CBTN data available for fine-tuning.


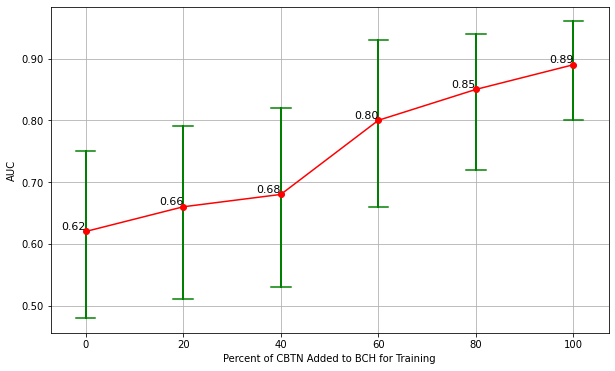


Figure S23. 3-year AUC with 95% confidence intervals for the multimodal survival model evaluated on the blinded CBTN test set with incrementally increasing CBTN data available for fine-tuning.

# Supplementary Figures


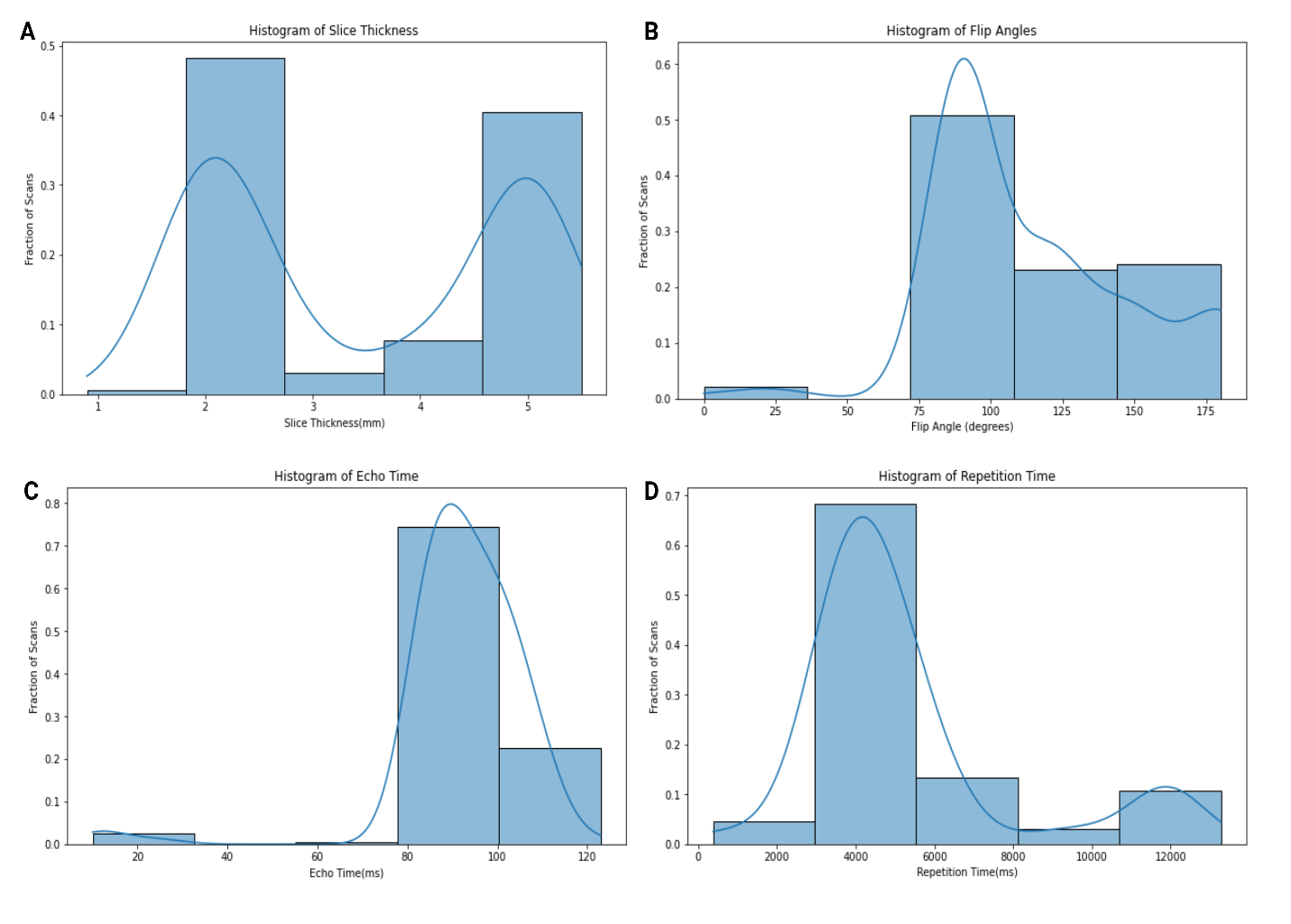


Figure S24. Histogram of MR parameters for DF/BCH cohort. (A) Slice Thickness, (B) Flip Angles, (C) Echo Time and (D) Repetition Time.


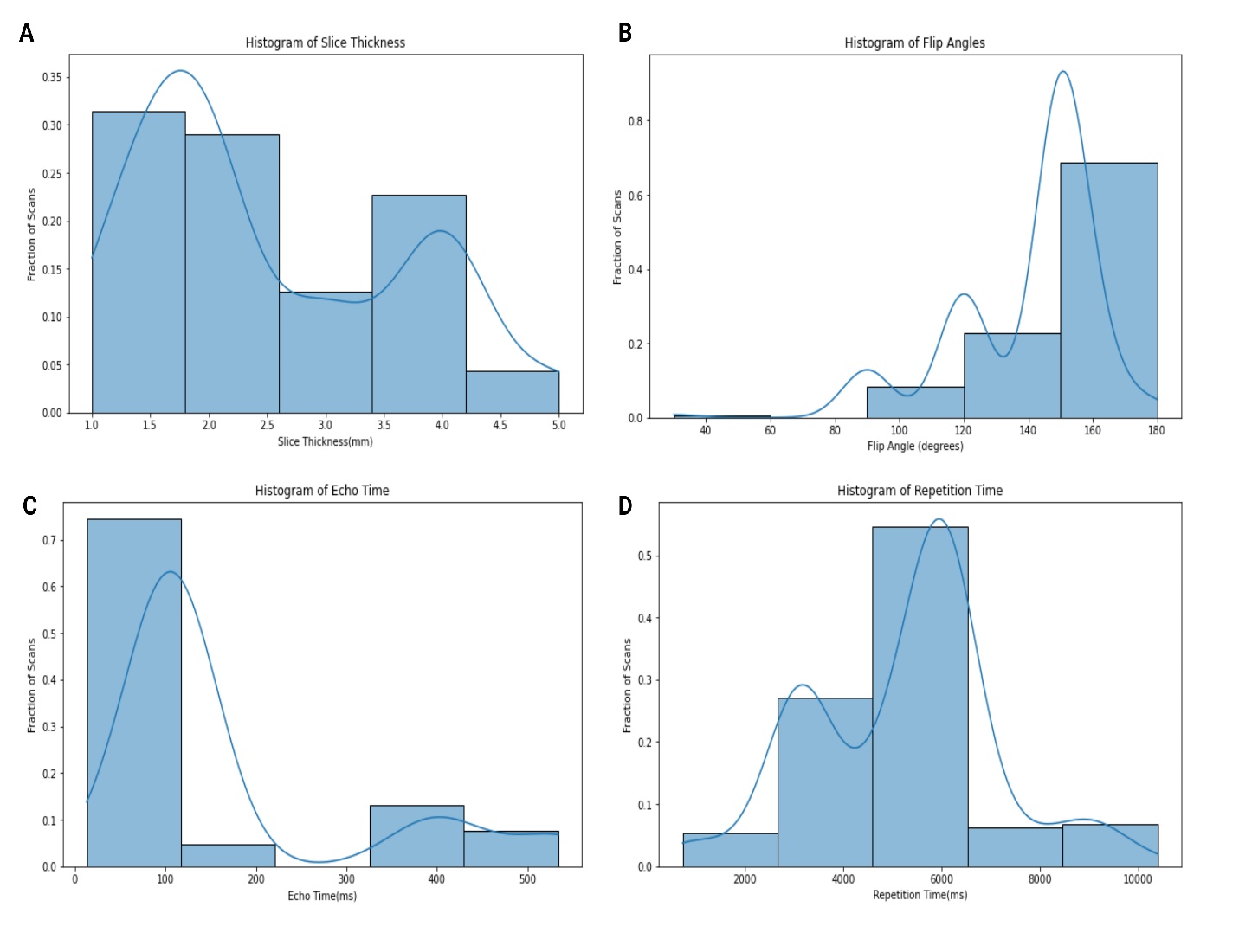
Figure S25. Histogram of MR parameters for CBTN cohort. (A) Slice Thickness, (B) Flip Angles, (C) Echo Time and (D) Repetition Time.


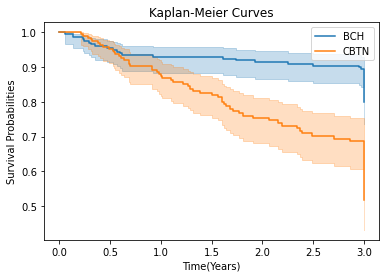


Figure S26. Three-Year Kaplan-Meier Survival Curves for DF/BCH and CBTN, Showing an EFS Rate of 89% for DF/BCH and 69% for CBTN.


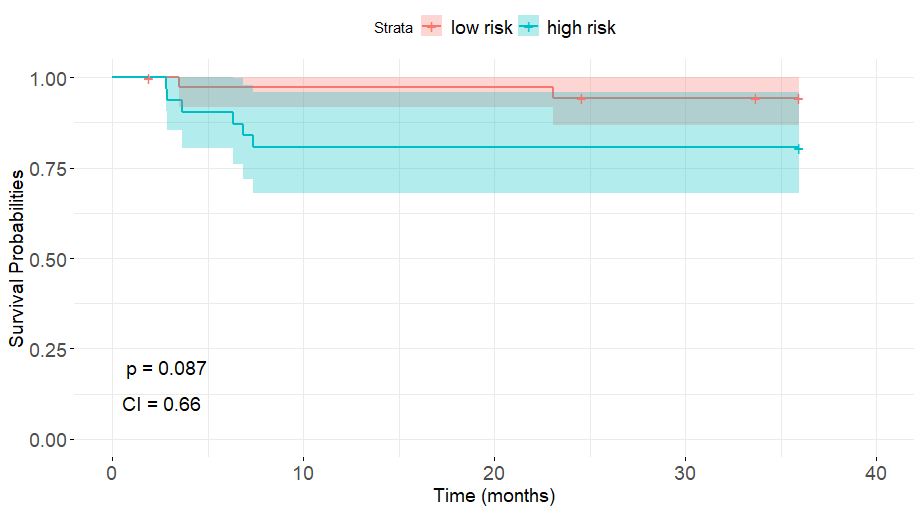


Figure S27. Kaplan-Meier Survival Curves for DF/BCH test subset: This figure illustrates the risk stratification of the clinical model, showcasing a 3-year EFS rate of 80% for the low-risk group and 94% for the high-risk group.


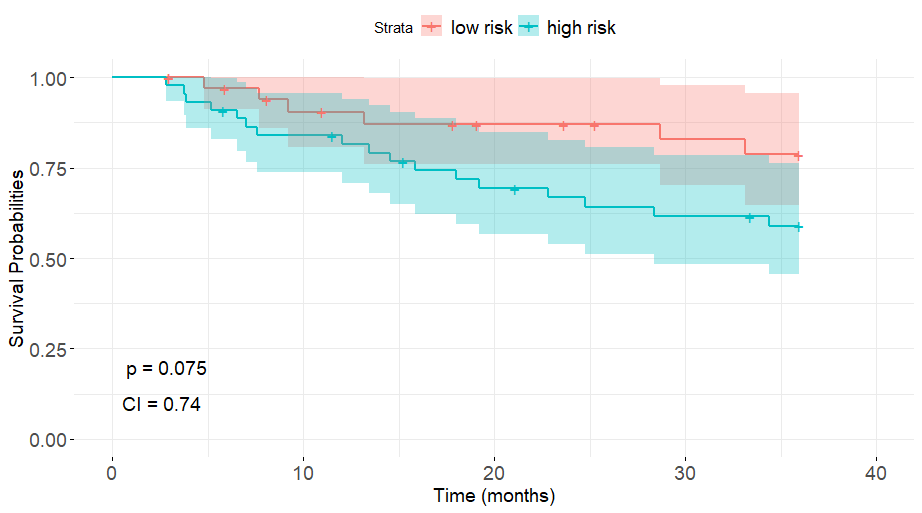


Figure S28. Kaplan-Meier Survival Curves for CBTN test subset: This figure illustrates the risk stratification of the clinical model, showcasing a 3-year EFS rate of 58% for the low-risk group and 78% for the high-risk group.


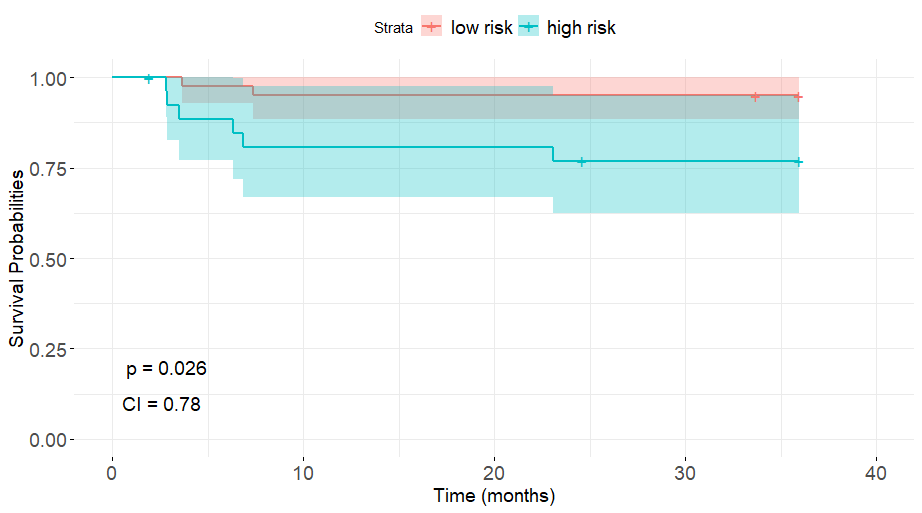


Figure S29. Kaplan-Meier Survival Curves for DF/BCH test subset: This figure illustrates the risk stratification of the DL-MRI model, showcasing a 3-year EFS rate of 76% for the low-risk group and 95% for the high-risk group.


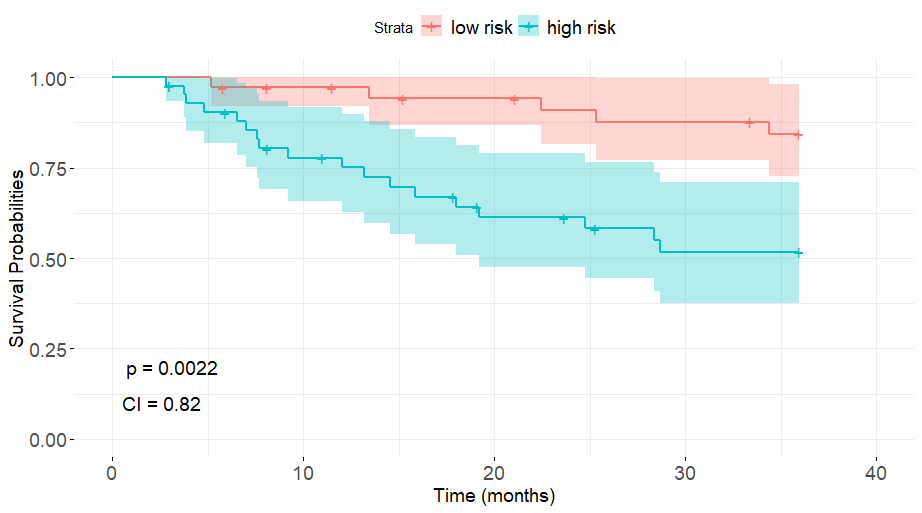


Figure S30. Kaplan-Meier Survival Curves for CBTN test subset: This figure illustrates the risk stratification of the DL-MRI model, showcasing a 3-year EFS rate of 51% for the low-risk group and 84% for the high-risk group.


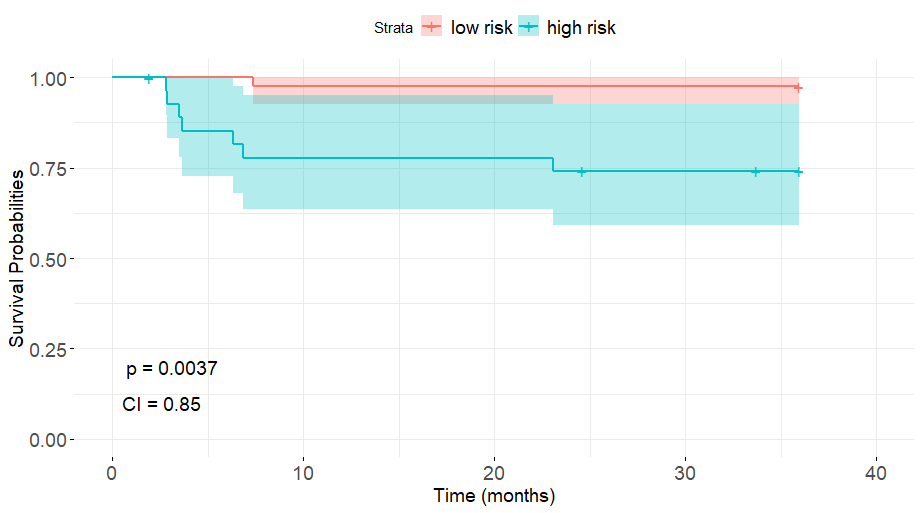


Figure S31. Kaplan-Meier Survival Curves for DF/BCH test subset: This figure illustrates the risk stratification of the multimodal model, showcasing a 3-year EFS rate of 74% for the low-risk group and 97% for the high-risk group.


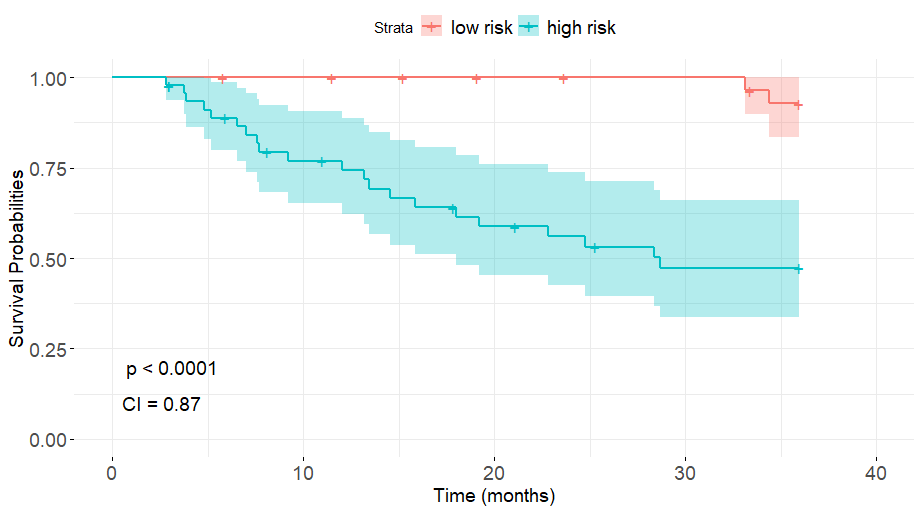


Figure S32. Kaplan-Meier Survival Curves for CBTN test subset: This figure illustrates the risk stratification of the multimodal model, showcasing a 3-year EFS rate of 47% for the low-risk group and 92% for the high-risk group.

# Supplementary Tables

Table S4. Comparative demographics of patients in the training and testing cohorts.

| Characteristics | Training cohort (N = 248) | Test cohort (N = 119) | p-value |
| --- | --- | --- | --- |
| Age (years) mean ± sd | [0.3,19.4], 6.5 | [0.5-19], 6.7 | 0.88 |
| Sex | | | |
| Male | 139 (56 %) | 60 (50 %) | 0.4 |
| Female | 109 (44 %) | 59 (50 %) |  |
| Resection Status | | | |
| Gross total | 132 (53 %) | 67 (56 %) | 0.71 |
| Partial | 79 (32 %) | 32 (27 %) |  |
| Biopsy | 36 (14 %) | 18 (15 %) |  |
| Not available | 1 (1 %) | 2 (2 %) |  |
| BRAF Mutational Status | | | |
| V600E | 18 (7%) | 12 (10%) | 0.32 |
| Fusion | 41(16%) | 22 (18%) |  |
| Wild | 29 (12%) | 9 (8%) |  |
| Unknown | 160 (65%) | 76 (64%) |  |
| BRAF Mutational Status (with unknowns inferred) | | | |
| V600E | 48 (19 %) | 23 (19 %) | 0.68 |
| Fusion | 93 (38 %) | 50 (42 %) |  |
| Wild | 107 (43 %) | 46 (39 %) |  |
| Chemotherapy | | | |
| Yes | 40 (16 %) | 14 (12 %) | 0.35 |
| No | 207 (83 %) | 104 (87 %) |  |
| Not Available | 1 (1 %) | 1 (1 %) |  |
| Radiotherapy | | | |
| Yes | 14 (6 %) | 6 (5 %) | 0.96 |
| No | 234 (94 %) | 111 (93 %) |  |
| Not Available | 0 (0 %) | 2 (2 %) |  |
| Tumor Location | | | |
| Posterior fossa | 98 (39 %) | 47 (39 %) | 0.75 |
| Supratentorial | 63 (25 %) | 37 (31 %) |  |
| Non-Cortical Supratentorial | 34 (14 %) | 16 (14%) |  |
| Optic Pathway/Non-Cortical Supratentorial | 15 (6 %) | 6 (5%) |  |
| Brainstem | 27 (11%) | 8 (7%) |  |
| Spinal Cord | 10 (4%) | 4 (3%) |  |
| Other | 1 (1%) | 1 (1%) |  |
| 3 year recurrence(N) | 43 (17 %) | 22 (18 %) | 0.88 |
| Total recurrence (N) | 68 (27 %) | 32 (27 %) | 0.9 |
| Recurrence time (days) range, median | [46, 8966], 1847 | [20, 7460], 1794 | 0.59 |

Table S5. Comparative MR parameters of patients in the DF/BCH and CBTN cohorts.

|  | DF/BCH | CBTN | p-value |
| --- | --- | --- | --- |
| Echo Time (milli second) range, median | (10.0, 123.0), 91.456 | (13.0, 534.0), 111.0 | <0.0001 |
| Repetition Time (milli second) range, median | (381.0, 13281.0), 4500 | (736.0, 10392.8), 5750 | 0.002 |
| Slice Thickness (milli meter) range, median | (0.90, 5.5), 3.0 | (1.0, 5.0), 2.0 | <0.0001 |
| Flip Angle (degree)  range, median | (0.0, 180.0), 90.0 | (30.0, 180.0), 150.0 | <0.0001 |

# References

1. Deudon, M. *et al.* HighRes-net: Recursive Fusion for Multi-Frame Super-Resolution of Satellite Imagery. Preprint at https://doi.org/10.48550/arXiv.2002.06460 (2020).

2. Tak, D. *et al.* Noninvasive molecular subtyping of pediatric low-grade glioma with self-supervised transfer learning. *medRxiv* 2023.08.04.23293673 (2023) doi:10.1101/2023.08.04.23293673.

3. Nobre, L. *et al.* Outcomes of BRAF V600E Pediatric Gliomas Treated With Targeted BRAF Inhibition. *JCO Precis. Oncol.* 561–571 (2020) doi:10.1200/PO.19.00298.

4. Lassaletta, A. *et al.* LG-46INFERIOR OUTCOME AND POOR RESPONSE TO CONVENTIONAL THERAPIES IN PEDIATRIC LOW-GRADE GLIOMAS HARBORING THE BRAF V600E MUTATION. *Neuro-Oncol.* **18**, iii89 (2016).

5. Bower, H. *et al.* Capturing simple and complex time-dependent effects using flexible parametric survival models: A simulation study. *Commun. Stat. - Simul. Comput.* **50**, 3777–3793 (2021).

6. Cox, D. R. Regression Models and Life-Tables. *J. R. Stat. Soc. Ser. B Methodol.* **34**, 187–220 (1972).

7. Longato, E., Vettoretti, M. & Di Camillo, B. A practical perspective on the concordance index for the evaluation and selection of prognostic time-to-event models. *J. Biomed. Inform.* **108**, 103496 (2020).

8. Lambert, J. & Chevret, S. Summary measure of discrimination in survival models based on cumulative/dynamic time-dependent ROC curves. *Stat. Methods Med. Res.* **25**, 2088–2102 (2016).

9. Austin, P. C., Harrell Jr, F. E. & van Klaveren, D. Graphical calibration curves and the integrated calibration index (ICI) for survival models. *Stat. Med.* **39**, 2714–2742 (2020).

10. Gerds, T. A. & Schumacher, M. Consistent estimation of the expected Brier score in general survival models with right-censored event times. *Biom. J. Biom. Z.* **48**, 1029–1040 (2006).
